# Rhein Suppresses Colorectal Cancer Cell Growth by Inhibiting the mTOR Pathway In Vitro and In Vivo

**DOI:** 10.3390/cancers13092176

**Published:** 2021-04-30

**Authors:** Haibo Zhang, Jun-Koo Yi, Hai Huang, Song Park, Sijun Park, Wookbong Kwon, Eungyung Kim, Soyoung Jang, Si-Yong Kim, Seong-Kyoon Choi, Sung-Hyun Kim, Kangdong Liu, Zigang Dong, Zae Young Ryoo, Myoung Ok Kim

**Affiliations:** 1 Department of Animal Science and Biotechnology, ITRD, Kyungpook National University, Sangju 37224, Korea; 2018326925@knu.ac.kr (H.Z.); huanghai@knu.ac.kr (H.H.); kge99777@knu.ac.kr (E.K.); 2 Gyeongbuk Livestock Research Institute, Yeongju 36052, Korea; 79lee38@korea.kr; 3 Core Protein Resources Center, DGIST, Daegu 41566, Korea; cristaling@dgist.ac.kr (S.P.); cskbest@dgist.ac.kr (S.-K.C.); 4 Department of Brain and Cognitive Science, DGIST, Daegu 41566, Korea; 5 School of Life Sciences, BK21 FOUR KNU Creative Bioresearch, Kyungpook National University, Daegu 41566, Korea; stemsj@knu.ac.kr (S.P.); jangsy@knu.ac.kr (S.J.); kim_si-yong@knu.ac.kr (S.-Y.K.); 6 Division of Biotechnology, DGIST, Daegu 41566, Korea; bongdaling@dgist.ac.kr; 7 Department of Bio-Medical Analysis, Korea Polytechnic College, Chungnam 34134, Korea; shkim92@kopo.ac.kr; 8 China-US (Henan) Hormel Cancer Institute, Zhengzhou 450008, China; kdliu@zzu.edu.cn (K.L.); zgdong@hi.umm.edu (Z.D.)

**Keywords:** Rhein, colorectal cancer, mTOR, xenograft

## Abstract

**Simple Summary:**

Colorectal cancer (CRC) is the fourth most common cancer and the second most common cause of cancer-related deaths globally. Rhein is a natural anthraquinone extract from rhubarb, which exhibits potent anticancer activity in various cancers. In this study, we show that rhein significantly inhibited the growth, migration, and invasion of CRC cells by directly binding to mTOR and inhibiting the mTOR signaling pathway. Rhein promotes mTOR degradation through the ubiquitin–proteasome pathway. In addition, rhein significantly suppressed tumor growth in a xenograft mouse model without obvious toxicity. Our results indicate that rhein is a promising anticancer agent that may be useful for the prevention and treatment of CRC.

**Abstract:**

Colorectal cancer (CRC) is one of the leading causes of mortality and morbidity in the world. Rhein has demonstrated therapeutic effects in various cancer models. However, its effects and underlying mechanisms of action in CRC remain poorly understood. We investigated the potential anticancer activity and underlying mechanisms of rhein in CRC in vitro and in vivo. Cell viability and anchorage-independent colony formation assays were performed to examine the antigrowth effects of rhein on CRC cells. Wound-healing and Transwell assays were conducted to assess cell migration and invasion capacity. Cell cycle and apoptosis were investigated by flow cytometry and verified by immunoblotting. A tissue microarray was used to detect mTOR expression in CRC patient tissues. Gene overexpression and knockdown were done to analyze the function of mTOR in CRC. The anticancer effect of rhein in vivo was assessed in a CRC xenograft mouse model. The results show that rhein significantly inhibited CRC cell growth by inducing S-phase cell cycle arrest and apoptosis. Rhein inhibited CRC cell migration and invasion through the epithelial–mesenchymal transition (EMT) process. mTOR was highly expressed in CRC cancer tissues and cells. Overexpression of mTOR promoted cell growth, migration, and invasion, whereas mTOR knockdown diminished these phenomena in CRC cells in vitro. In addition, rhein directly targeted mTOR and inhibited the mTOR signaling pathway in CRC cells. Rhein promoted mTOR degradation through the ubiquitin-proteasome pathway. Intraperitoneal administration of rhein inhibited HCT116 xenograft tumor growth through the mTOR pathway. In conclusion, rhein exerts anticancer activity in vitro and in vivo by targeting mTOR and inhibiting the mTOR signaling pathway in CRC. Our results indicate that rhein is a potent anticancer agent that may be useful for the prevention and treatment of CRC.

## 1. Introduction

Colorectal cancer (CRC) is the third most commonly diagnosed cancer and the second leading cause of cancer-related deaths among men and women combined in the United States [1]. The incidence and mortality of CRC has risen steadily around the world. Surgery remains the most effective treatment option for CRC, but the risk of recurrence is high [2,3]. Thus, there is an urgent need for the development of more effective chemotherapy for CRC.

The progression of CRC is accompanied by gene mutations and abnormal gene expression [4,5]. The PI3K/Akt/mTOR pathway is commonly deregulated in human cancer. mTOR is considered as a major regulator of this signaling pathway, which regulates multiple biological processes including cell proliferation, autophagy, and inflammation [6]. Deregulation of the mTOR signaling pathway is also involved in tumor etiology and progression [7,8]. Targeting mTOR signaling has generated significant interest for cancer therapy [9,10]. In fact, mTOR inhibitors, such as temsirolimus and everolimus, have been approved for the treatment of breast and renal cancer [10]. In CRC, tandutinib [11] and tunicamycin [12] were reported to inhibit colon cancer growth by suppressing the mTOR signaling pathway. Therefore, targeting mTOR is a promising strategy for developing novel anticancer therapeutics.

Natural compounds represent a major source for drug development [13,14,15]. Rhein (4,5-dihydroxyanthraquinone-2-carboxylic acid) is a natural anthraquinone found in several medicinal plants [16], particularly in rhubarb [17]. As a traditional Chinese medicine, rhubarb has been used medicinally in China for thousands of years and it regulates gastrointestinal, anticancer, antimicrobial, hepatoprotective, and anti-inflammatory properties [18]. Previous studies have suggested that rhein inhibits non-small cell lung cancer cell growth in vitro and in vivo by suppressing the STAT3 signaling pathway [19] and inducing HepaRG cell death through S-phase cell cycle arrest and apoptosis [20]. In addition, anticancer activity of rhein has been observed in breast [21], ovarian [22], and colon [23] cancers, suggesting that it may be a novel agent for the prevention and treatment of CRC.

Although previous studies suggest that rhein exhibits potent anticancer activity, only two studies have been reported showing that rhein inhibits the growth of CRC cells and enhances the effect of lymphocytes [23] and erlotinib [24] in CRC. The mechanism of action of rhein in CRC is still largely unknown and the direct target proteins of rhein have not been identified. In this study, we investigated that rhein suppresses CRC cell growth by inhibiting mTOR signaling pathway in vitro and in vivo, and rhein promotes mTOR degradation through the ubiquitination–proteasome pathway. Our results reveal that rhein may be a potential candidate for CRC treatment.

## 2. Materials and Methods

### 2.1. Reagents and Antibodies

Rhein (purity: >98% assessed by HPLC) was purchased from Harvey Biotech Co., Ltd. (Beijing, China). For in vitro experiments, rhein was dissolved in dimethyl sulfoxide (DMSO). CNBr Sepharose 4B beads were obtained from GE Healthcare (Piscataway, NJ, USA). The primary antibodies PI3K, p-Akt, Akt, p-mTOR, mTOR, cleaved caspase3, p53, p-p53, Bax, p70 S6 Kinase (p70S6K), p-p70 S6 Kinase (p-p70S6K), cyclin-dependent kinase (CDK) 2, cyclin D1, cyclin E1, E-cadherin, N-cadherin, Heat shock protein 90 (HSP90), 4EBP1, and p-4EBP1 were purchased from Cell Signaling Technology (Beverly, MA, USA). β-Actin, Heat shock factor 1 (HSF1), and cyclin A1 were purchased from Santa Cruz Biotechnology (Santa Cruz, CA, USA). Ki-67 and vimentin were purchased from Abcam (Cambridge, MA, USA).

### 2.2. Cell Culture

The human CRC cell lines HCT15, HCT116, DLD1, HT29, SW620, and CCD-18Co normal human colon fibroblasts cells were purchased from the American Type Culture Collection (ATCC). HCT116 and HT29 cells were cultured in McCoy’s 5A medium. SW620 cells were cultured in L15 medium (Leibovitz). HCT15 and DLD1 were cultured in RPMI1640 medium. CCD-18Co cells were cultured in MEM medium (Leibovitz). All medium was supplemented with 10% FBS (Gibco) and 1% antibiotics. All cells were incubated at 37 °C in a 5% CO_2_ humidified incubator. All the cells were cytogenetically tested and authenticated before being frozen. Each vial of frozen cells was thawed and maintained in culture for a maximum of 8 weeks.

### 2.3. Cell Viability Assay

CRC cells were seeded into 96-well plates (1 × 10^3^ cells/well) to allow attachment, incubated overnight, then treated with various concentrations of rhein or DMSO for 0, 24, 48, and 72 h. Next, 10 µL of CCK-8 solution (Dojindo Japan) was added to each well and the plates were incubated for an additional 1 h at 37 °C in a 5% CO_2_ incubator. The absorbance at 450 nm was measured using a spectrophotometer (BioTek).

### 2.4. Anchorage-Independent Cell Growth 

CRC cells (8 × 10^3^ cells/well) were seeded into complete growth medium containing 0.3% agar with various concentrations of rhein, then overlaid into a 6-well plate containing a 0.6% agar base and various concentrations of rhein. Plates were incubated at 37 °C in a 5% CO_2_ incubator for 2 weeks, then photographed under a microscope (Leica, Wetzlar, Germany) and the resulting colonies were counted using ImageJ software.

### 2.5. Cell Cycle and Apoptosis Analysis

CRC cells were seeded into 60-mm culture dishes (2 × 10^5^ cells/dish). After incubating for 12 h, the cells were exposed to various concentrations of rhein for 48 h. For cell cycle assays, the cells were collected and fixed in 70% cold ethanol and stored at −20 °C overnight. The cells were stained using RNase (100 μg/mL) and propidium iodide (PI, 20 μg/mL) staining buffer. For apoptosis assays, the cells were collected and then stained with Annexin V (BioLegend, San Diego, CA, USA) and PI. The cell cycle and apoptosis were analyzed by flow cytometry (FACS Verse; BD Science, CA, USA).

### 2.6. Wound-Healing Assay

The migration of CRC cells was evaluated in wound-healing assays. When cells grew to 90% confluence in 6-well dishes, a scratch was created by scratching the monolayer with a 200 µL plastic pipette tip. Following treatment with various concentrations of rhein for 24 h, the cells were examined by microscopy at time 0, 12, and 24 h. The remaining wound area was determined using the ImageJ software (v. 4) program.

### 2.7. Migration and Invasion Assays

The Matrigel migration assay was performed using Transwell chambers (8 µm pore-size, Corning Inc., Corning, NY, USA) according to the manufacturer’s instructions. The lower compartment was filled with 650 μL medium containing 10% FBS. Cells (8 × 10^4^) in 100 μL medium containing 1% FBS were added to the upper chamber and 12 h later, they were treated with various concentrations of rhein in the upper chamber. After 48 h, the cells were fixed with 4% paraformaldehyde for 20 min and the noninvasive cells were removed using a cotton swab. The invading cells were stained with 0.1% crystal violet. For the invasion assays, the chamber was precoated with Matrigel^®^ and 8 × 10^4^ cells were seeded into the upper chamber. The other procedures were performed in the same manner as the migration assay. The invading cell numbers were quantified by counting the stained cells by microscopy. 

### 2.8. In Vitro Pull-Down Assay

To verify the interaction between rhein and mTOR, HCT15 and HCT116 cell lysates (500 μg) were incubated with Sepharose 4B or rhein-Sepharose 4B beads in a reaction buffer (150 mM NaCl, 50 mM Tris pH 7.5, 5 mM EDTA, 0.01% NP40, 1 mM DTT, 2 μg/mL Bovine serum albumin). After gentle rocking overnight at 4°C, the beads were washed five times with wash buffer (50 mM Tris pH 7.5, 5 mM EDTA, 150 mM NaCl, 1 mM DTT, 0.01% NP40) and binding was visualized by Western blot analysis.

### 2.9. Protein Stability and Ubiquitination

To examine mTOR protein stability, HCT116 cells were seeded into 100-mm dishes and treated with cycloheximide (CHX, 10 μM) and MG132 (15 μM) at the indicated time points. To assess ubiquitination, HCT116 cells were transfected with the pcDNA-Flag-Ub plasmid. After 24 h of transfection and treatment with rhein (40 μM), the indicated dishes were treated with CHX (10 μM) and MG132 (15 μM) for 3 h before harvest. The cells were lysed with lysis buffer (50 mM Tris-HCl, pH 7.4, 150 mM NaCl, 1% NP-40, 1 mM EDTA) containing a protease inhibitor cocktail (Sigma) and protein extracts (1 mg) were incubated with anti-Flag antibody (M2) and rocked for 4 h at 4 °C. Dynabeads^®^ Protein G (30 μL) (Invitrogen, Carlsbad, CA, USA) was added to each tube and rocked for an additional 4 h. The beads were collected by a magnet and washed with wash buffer, resuspended in 50 μL of 1 × protein sample buffer, heated to 95 °C for 5 min, and ubiquitination was analyzed by Western blot analysis.

### 2.10. Establishment of Stable mTOR Overexpressing Cell Lines

pcDNA3-Flag mTOR wt was a gift from Jie Chen (Addgene # 26603) [25]. The empty vector (pcDNA3.1) was purchased from Invitrogen (V79020). For transfection experiments, FuGENE HD transfection reagent (Promega) was used following the manufacturer’s instructions. For stable transfection, the cells were treated with 600 μg/mL of G418 (Gibco) for 2 weeks. Individual G418-resistant cells were maintained in the presence of G418.

### 2.11. Lentiviral Production and Infection

The lentiviral mTOR shRNA vectors (sh-mTOR#2 sequence: 5′-CCGG CCTG GCAA CAATAGGAGAATTCTCGAGAATTCTCCTATTGTTGCCAGGTTTTTG-3′; sh-mTOR#3 sequence: 5′-CCGGGCAACCCTTCTTTGACAACATCTCGGATGTTGTCAAAGAAGGG

TTGCTTTTTG-3′) for knockdown of mTOR were purchased from Sigma and 293T cells were cotransfected with pLKO.1-mock or pLKO.1-sh-mTOR and the packaging vectors (pMDLg/pRRE, pMD2.G, and pRSV-Rev) using transfection reagent (FuGENE HD, Promega, Madison, WI, USA). Viral particles were collected by filtration using a 0.45-μm filter and stored at −80 °C. The cultured CRC cells were infected with virus particles together with 8 μg/mL polybrene (Millipore, Billerica, MA, USA) for 24 h. The medium was replaced and selected with puromycin (2 μg/mL) for 48 h. The effects of mTOR knockdown on CRC cells were determined in subsequent experiments.

### 2.12. Western Blotting Assay

Protein extracts were prepared from cells and tumor tissues using PRO-PREP™ lysis buffer (Intron Biotechnology, Seongnam, Korea). The protein concentrations were measured with a NanoDrop™ 2000 (Thermo Fisher Scientific, Wilmington, DE, USA) instrument. Protein obtained from the lysates were separated by SDS-PAGE and transferred onto polyvinylidene difluoride membranes (0.22 µm, Merck Millipore, Durapore, Darmstadt, Germany). The membranes were incubated overnight with the primary antibodies at 4 °C. Subsequently, the membranes were incubated with the corresponding secondary antibodies for 1 h at room temperature. Immunoblots were developed using an ECL detection kit (GE Healthcare, Seoul, Korea) and visualized using a Da Vinci Fluorescence Imaging System (Da Vinci-K, Seoul, Korea). β-Actin was used as a loading control.

### 2.13. Immunofluorescence Analysis

CRC cells were seeded into 2-well plates and treated with various concentrations of rhein for 48 h. The cells were then fixed in 4% formaldehyde for 15 min, permeabilized with 0.3% Triton X-100, and incubated with mTOR antibody (1:500; Cat # 2983 CST) overnight at 4 °C. The secondary antibody, Alexa Fluor 488-conjugated goat anti-rabbit IgG antibody (Invitrogen), was incubated with the cells at room temperature for 1 h in the dark. The nuclei were stained with 4′, 6-diamidino-2-phenylindole (DAPI). Representative images were obtained using a fluorescence microscope (Leica).

### 2.14. In Vivo Xenograft Experiments

All animal experiments were performed according to the guidelines and approval of Kyungpook National University (No.2015-0135). Male athymic nude mice (4~5 weeks) were purchased from Charles River Technology (Boston, MA, USA) through Orient Bio Inc. (Sungnam, Gyeonggi, Korea). Food and water were provided ad libitum. To establish CRC xenografts, HCT116 cells (1 × 10^7^ cells) suspended in 200 μL PBS were subcutaneously injected into the flanks of the mice. Six days following implantation, the mice were randomly divided into three groups (*n* = 8 mice/group). Two groups were treated with rhein at 10 or 50 mg/kg body weight (dissolved in 5% Dimethyl sulfoxide (DMSO) and 10% Tween-20 in PBS), whereas the third group was treated with vehicle only. Rhein or vehicle was intraperitoneally injected three times a week for 32 days. Tumor volumes and body weights were measured every 4 days. Tumor volume was calculated using the following ellipsoid formula: tumor volume (mm^3^) (length × width × height × 0.52). 

### 2.15. Immunohistochemical Staining

The tumor tissues sections were baked at 60 °C overnight, rehydrated with xylene and graded alcohols. Antigen retrieval was done by heat treatment in citrate buffer (pH 6.0). Primary antibodies were incubated with the sections overnight at 4 °C (Ki-67, 1:200; cyclin D1, 1:200; HSF1, 1:100; cyclin A1, 1:200), followed by incubation with biotin-conjugated secondary antibody for 1 h at 37 °C. Images were obtained by microscopy and analyzed using the ImageJ software (v. 4) program. 

### 2.16. Tissue Microarray

A sample consisting of 70 pairs of colorectal cancer tissues and adjacent correspondent tissues was collected from patients who had not received chemotherapy or radiation treatment during surgery at the Huashan Hospital Fudan University in 2019. These samples were paraffin-embedded by the Service Biocompany (Shanghai, China). The study was approved by the Committee for the Ethical Review of Research Involving Human Subjects at the Huashan Hospital of Fudan University and written informed consent was obtained from each participant in this study. 

### 2.17. Statistical Analysis

All data were presented as the mean ± SD from at least three independent experiments. Statistical significance was determined using a Student’s *t*-test. A *p* value < 0.05 was considered statistically significant.

## 3. Results

### 3.1. Rhein Inhibits the Growth of CRC Cells

**Figure 1:**
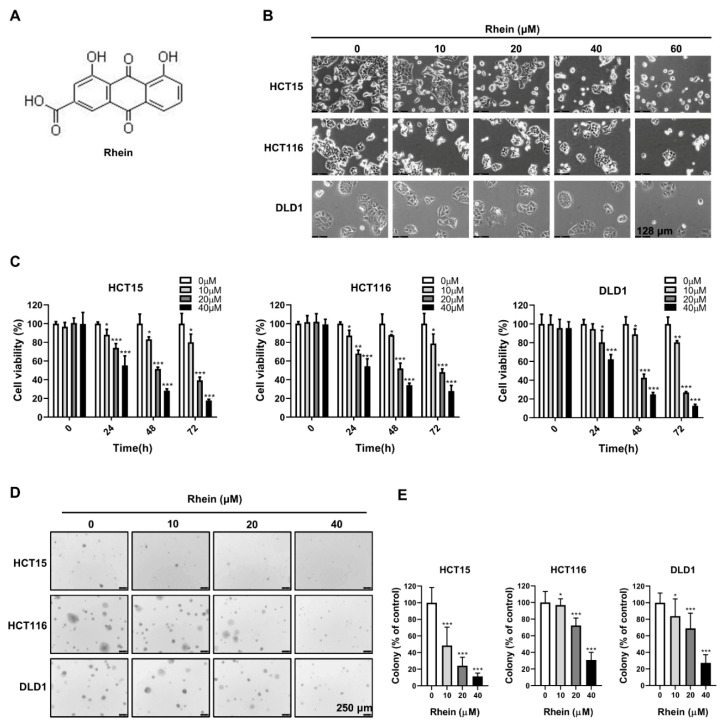
Rhein inhibits the growth of CRC cells. (**A**) Chemical structure of rhein. (**B**) Morphological changes of HCT15, HCT116, and DLD1 cells following treatment with 0, 10, 20, 40, and 60 μM rhein for 24 h (Scale bar = 128 μm). (**C**) HCT15, HCT116, and DLD1 cells were treated with rhein (0, 10, 20, and 40 μM) for 0, 24, 48, and 72 h. Cell viability was determined using the CCK-8 assay. (**D**,**E**) Effect of rhein on anchorage-independent growth of CRC cells (Scale bar = 250 μm). * *p* < 0.05, ** *p* < 0.01, *** *p* < 0.001.

The chemical structural of rhein is shown in Figure 1A. To explore the effect of rhein on CRC cell growth, HCT15, HCT116, and DLD1 cells were treated with various concentrations of rhein (0, 10, 20, 40, and 60 μM) for 24 h. As shown in Figure 1B, there was significant cell death accompanied by morphological changes including round-shaped and transparent CRC cells at concentrations of 40 and 60 μM. Next, we evaluated the half-maximal inhibitory concentration (IC_50_) of rhein in HCT15, HCT116, and DLD1 cells. The resulting IC_50_ values were 41.25 μM, 47.77 μM, and 46.51 μM, respectively, at 24 h (Figure S1), whereas the viability of CCD-18Co normal colon fibroblasts cells was unaffected by the rhein treatment (Figure S1). Therefore, concentrations of 0, 10, 20, and 40 μM were selected for subsequent experiments. The results of a CCK-8 assay indicated that rhein significantly suppressed proliferation of HCT15, HCT116, and DLD1 cells in a dose-dependent manner (Figure 1C). The results of the anchorage-independent colony formation assay revealed a significant decrease in colony number following rhein treatment (Figure 1D,E). Collectively, rhein effectively inhibited CRC cell growth and was less cytotoxic to normal colon fibroblasts cells.

### 3.2. Rhein Inhibits the Migration and Invasion of CRC Cells

**Figure 2:**
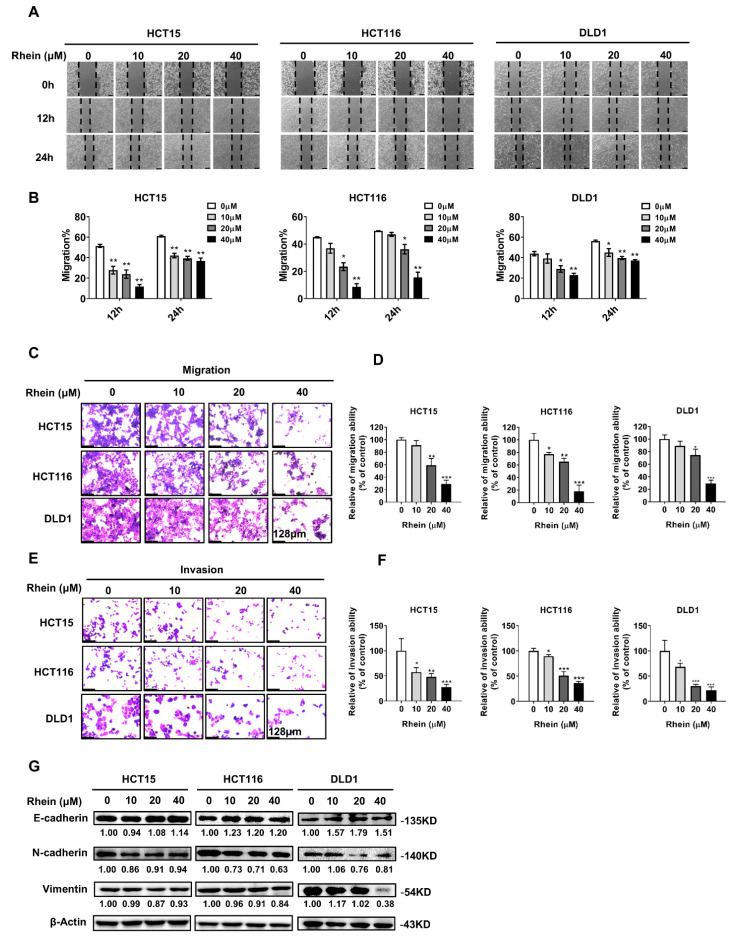
Rhein inhibits the CRC cells migration and invasion. (**A**) A wound-healing assay was performed to evaluate cell migration in HCT15, HCT116, and DLD1 cells. (**B**) Quantitative analysis of the migration of HCT15, HCT116, and DLD1 cells. (**C**) Representative images of migrating cells in HCT15, HCT116, and DLD1 cells. (**D**) Cell migration presented as a percentage of the control. (**E**) Representative images of invading cells representing HCT15, HCT116, and DLD1 cells. (**F**) Cell invasion presented as a percentage of the control. (**G**) Western blot analysis of EMT-related proteins in HCT15, HCT116, and DLD1 cells treated with rhein compared with the control. Scale bar = 128 μm. * *p* < 0.05, ** *p* < 0.01, *** *p* < 0.001. Uncropped western blots figures are shown in Figure S2.

Cancer metastasis is the primary cause of cancer death from solid tumors [26]. To determine whether rhein inhibits the migration and invasion of CRC cells, we first determined the effect of rhein on the motility of CRC cells using a wound-healing assay. HCT15, HCT116, and DLD1 cells were treated with rhein at 0, 10, 20, and 40 μΜ for 24 h. The results demonstrated that cell motility was inhibited by rhein treatment in HCT15, HCT116, and DLD1 cells compared with that in control cells at 12 h or 24 h (Figure 2A,B). Next, the effect of rhein on CRC cell migration and invasion was measured by Transwell assay. After treatment with rhein (0, 10, 20, or 40 μM) for 48 h, the number of migrating or invading cells was significantly decreased in a dose-dependent manner (Figure 2C–F). It is known that the epithelial–mesenchymal transition (EMT) is closely associated with cancer cell migration and invasion [27]. To determine the role of rhein in the EMT process, we examined the expression of several EMT-related proteins including E-cadherin, N-cadherin, and vimentin using Western blot assays. As expected, treatment with rhein upregulated the expression of E-cadherin and downregulated the expression of N-cadherin and vimentin (Figure 2G). These findings suggest that rhein significantly inhibits CRC cell migration and invasion by regulating the expression of EMT-related proteins.

### 3.3. Rhein Induces S-Phase Cell Cycle Arrest and Apoptosis of CRC Cells

**Figure 3:**
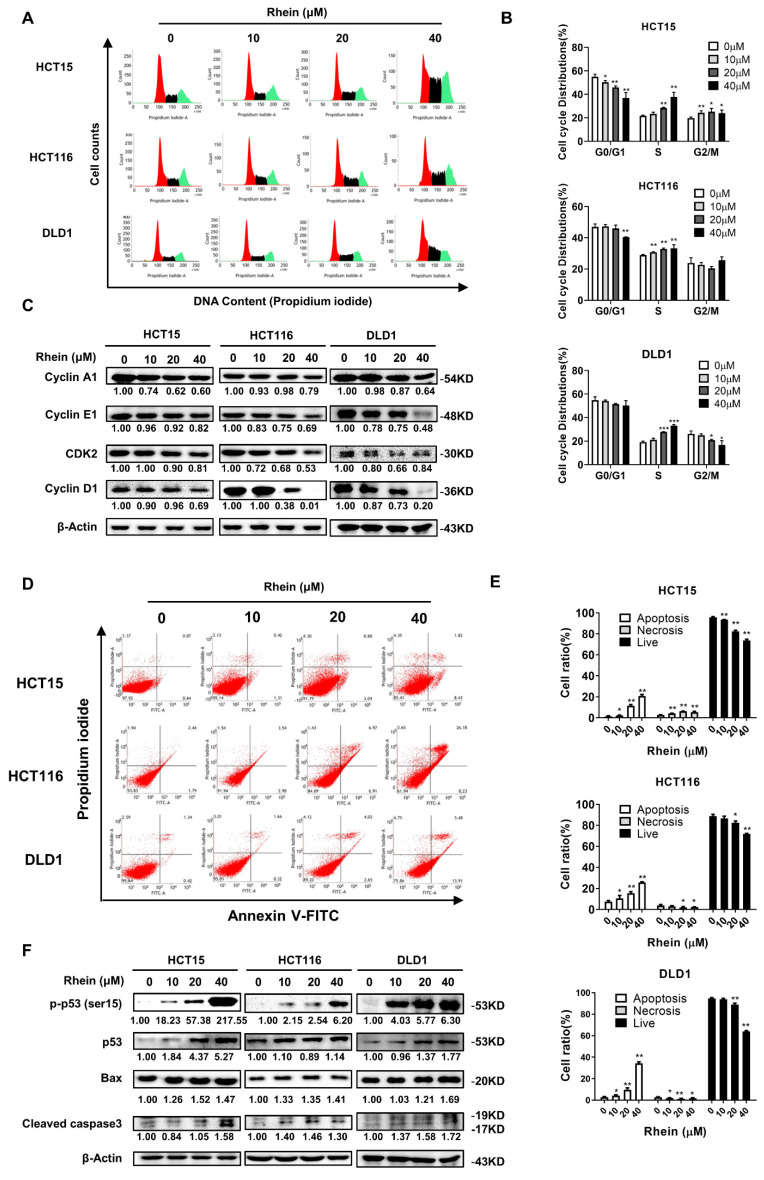
Rhein induces S-phase cell cycle arrest and apoptosis in CRC cells. (**A**,**B**) Flow cytometry was used to determine the cell cycle distribution of rhein-treated CRC cells. Cells were treated with 0, 10, 20, and 40 μM rhein for 48 h and cell cycle was analyzed by flow cytometry. (**C**) The expression of cyclin A1, cyclin E1, cyclin D1, and CDK2 in CRC cells were measured by Western blot analysis. (**D**,**E**) Cells were treated with 0, 10, 20, or 40 μM rhein for 48 h and apoptosis was assessed by flow cytometry. (**F**) Expression of p-p53, p53, Bax, and cleaved caspase-3 proteins were measured by Western blot analysis. * *p* < 0.05, ** *p* < 0.01, *** *p* < 0.001. Uncropped western blots figures are shown in Figure S2.

To further explore the effect of rhein on the proliferation of CRC cells, we investigated cell cycle distribution after 48 h treatment with rhein in CRC cells by flow cytometry. The results indicated that rhein treatment markedly increased the number of CRC cells arrested in S-phase (Figure 3A,B). To confirm this change, we examined the levels of S-phase regulatory proteins by Western blot analysis. The results showed that rhein treatment significantly decreased the expression of cyclin A1, cyclin E1, and CDK2 in CRC cells (Figure 3C). The mTOR pathway has been reported to regulate the translation of cyclin D1 [28]. Therefore, we examined whether rhein treatment can influence the expression of cyclin D1 and found that the expression of cyclin D1 was downregulated (Figure 3C). These results suggest that rhein-induced S-phase cell cycle arrest by downregulating the expression of cyclin A1, cyclin E1, CDK2, and cyclin D1. We next examined whether rhein induces apoptosis of CRC cells using Annexin V/PI staining and flow cytometry. The results demonstrated that rhein-induced apoptosis in HCT15, HCT116, and DLD1 cells in a dose-dependent manner (Figure 3D,E). In addition, the apoptotic marker proteins p53, p-p53, cleaved caspase 3, and Bax were upregulated in CRC cells after treatment with rhein for 48 h (Figure 3F). 

### 3.4. Rhein Directly Targets mTOR and Suppresses mTOR Signaling in CRC Cells

**Figure 4:**
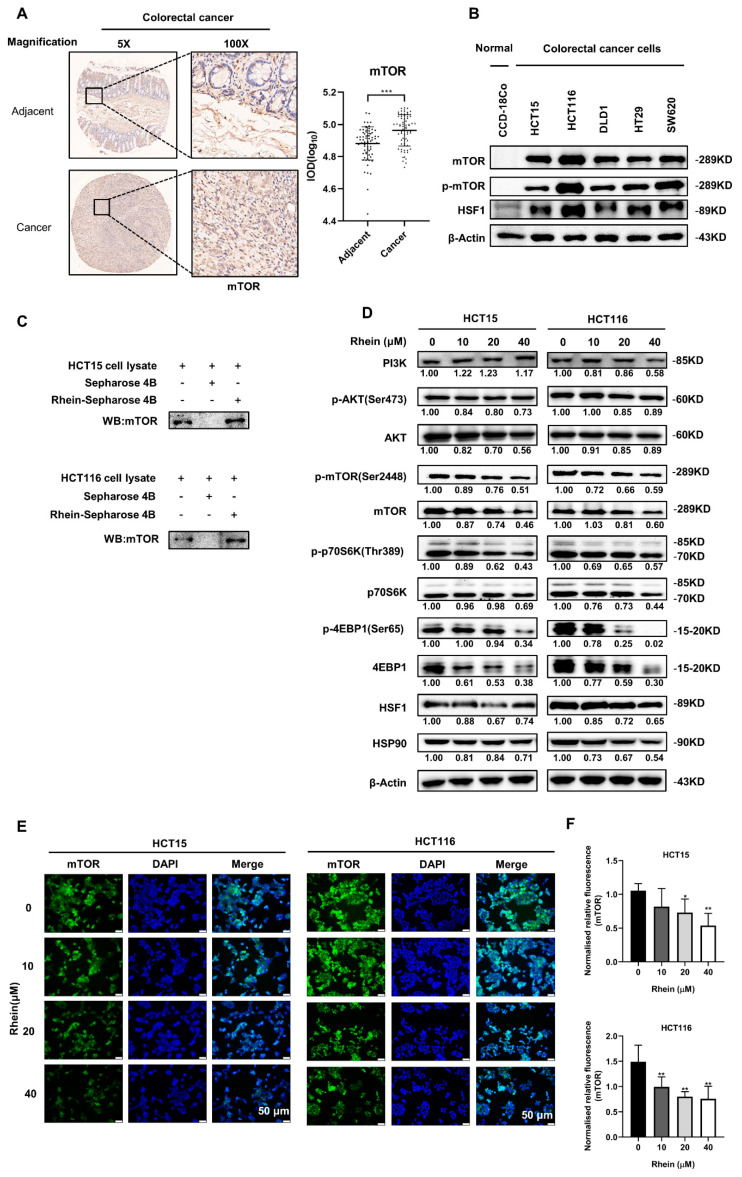
Rhein directly targets mTOR and suppresses the mTOR signaling pathway in CRC cells. (**A**) The expression of mTOR was evaluated by IHC analysis using a CRC tumor microarray. (**B**) mTOR, p-mTOR, and HSF1 expression levels in CRC cell lines and the CCD-18Co normal colon cell line were measured by Western blot analysis. (**C**) The binding of rhein to mTOR in HCT15 and HCT116 cell lysates was determined by Western blot analysis. (**D**) The effects of rhein (48 h) on the mTOR signaling pathway in CRC cells were determined by Western blot analysis. (**E**,**F**) Immunofluorescence results showed that mTOR levels are decreased after rhein treatment for 48 h compared with the control (Scale bars = 50 μm). * *p* < 0.05, ** *p* < 0.01, *** *p* < 0.001. Uncropped western blots figures are shown in Figure S2.

Studies have shown that high mTOR expression is associated with poor prognosis [29,30]. An immunohistochemical study performed in 154 patients indicated that p-mTOR (Ser2448) and p-p70S6K (Thr389) were overexpressed in CRC tumor tissues compared to in normal colon tissues [31]. In the present study, we measured the expression of mTOR using a CRC tumor microarray that included 70 pairs of cancer tissues and adjacent normal tissues (Figure 4A). We found that mTOR was significantly overexpressed in the cancer tissues compared with that in the adjacent normal tissues (Figure 4A). We then evaluated mTOR expression in CRC cell lines and found that mTOR was highly expressed in CRC cell lines, especially in HCT15 and HCT116 cells, compared with CCD-18Co cells (Figure 4B). These results suggest that mTOR is a potential therapeutic target for CRC treatment. In addition, p-mTOR and HSF1 were highly expressed in CRC cells compared with CCD-18Co cells (Figure 4B). A previous study reported that rhein induces apoptosis through the PI3K/AKT/mTOR signaling pathway in A549 human lung cancer cells [32] and inhibits autophagy by regulating AMPK/mTOR signaling in rat renal tubular cells [33]. To determine whether rhein can directly target mTOR protein, we performed in vitro pull-down assays using rhein-conjugated Sepharose 4B beads (or Sepharose 4B beads only as a negative control) and HCT15 or HCT116 cell lysates (Figure 4C). Immunoblotting results demonstrated that rhein was directly bound to the mTOR protein (Figure 4C). We then investigated the effect of rhein on mTOR signaling in CRC cells. The results showed that treatment with rhein downregulated p-mTOR, p-p70S6K, and p-4EBP1 in both HCT15 and HCT116 cells (Figure 4D). HSF1 activation in multiple cancers is significantly associated with tumor metastasis and death [34] and mTOR is essential for HSF1 activation and HSP90 synthesis [35]. Therefore, we measured HSF1 and HSP90 expression following treatment with rhein in HCT15 and HCT116 cells. As expected, the protein levels of HSF1 and HSP90 were downregulated by rhein treatment in the CRC cells (Figure 4D). The results of immunofluorescence analysis showed that mTOR expression was suppressed after rhein treatment compared with that in the control (Figure 4E,F). These results indicate that rhein directly targets mTOR and inhibits the mTOR signaling pathway in CRC cells.

### 3.5. Rhein Promotes mTOR Protein Degradation by the Ubiquitin-Proteasome Pathway

**Figure 5:**
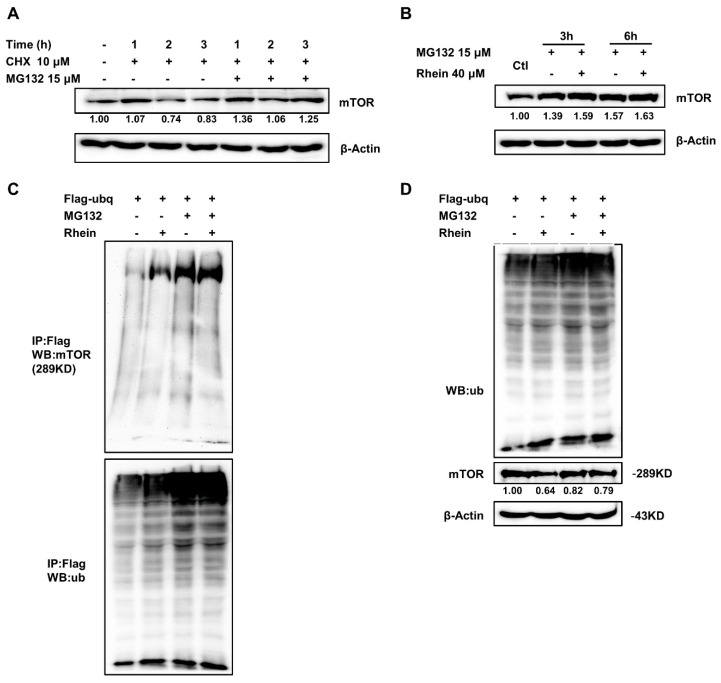
Rhein promotes mTOR protein degradation by the ubiquitin–proteasome pathway. (**A**) HCT116 cells were treated with cycloheximide (CHX; 10 μM) and MG 132 (15 μM) for 1, 2, and 3 h and mTOR protein expression was measured by Western blot analysis. (**B**) HCT116 cells were treated with MG132 in the absence or presence of 40 μM rhein for 3 or 6 h and mTOR protein expression was measured by Western blot analysis. (**C**,**D**) HCT116 cells were transfected with Flag-tagged ubiquitin plasmid for 24 h followed by treatment with MG132 alone or in combination with 40 μM rhein. Protein ubiquitination was analyzed by Western blot analysis. Uncropped western blots figures are shown in Figure S2.

Since rhein can directly bind to mTOR and downregulates mTOR expression, we reasoned that rhein treatment might be attributed to the stability and degradation of the mTOR protein. It is well known that the ubiquitin–proteasome pathway plays an important role in protein degradation [36]. Moreover, studies have reported that mTOR can be degraded by ubiquitin-proteasome pathways [37,38,39]. To further explore the mechanism through which rhein regulates mTOR in CRC cells, we analyzed mTOR protein stability by treatment with cycloheximide (CHX, a protein synthesis inhibitor) and pretreated HCT116 cells for 1, 2, and 3 h in the presence or absence of the proteasome inhibitor, MG132 (15 μM). We found that CHX treatment rapidly reduced mTOR protein levels and cotreatment with MG132 restored mTOR protein levels (Figure 5A). Next, we treated cells with MG132 with or without rhein for 3 and 6 h. We found that MG132 treatment at 3 or 6 h significantly increased mTOR protein levels and the combination with rhein did not reduce mTOR protein levels, indicating that rhein downregulates mTOR expression by promoting its degradation (Figure 5B). To confirm the effect of ubiquitination on the degradation of mTOR expression, we overexpressed pcDNA-Flag-Ub plasmid in HCT116 cells and observed the ubiquitination of mTOR following rhein treatment. As shown in Figure 5C,D, rhein promoted the ubiquitination of mTOR. These findings indicated that rhein promotes mTOR protein degradation by the ubiquitin–proteasome pathway.

### 3.6. Overexpression of mTOR Promotes the Proliferation, Anchorage-Independent Colony Formation, Migration, and Invasion of CRC Cells

**Figure 6:**
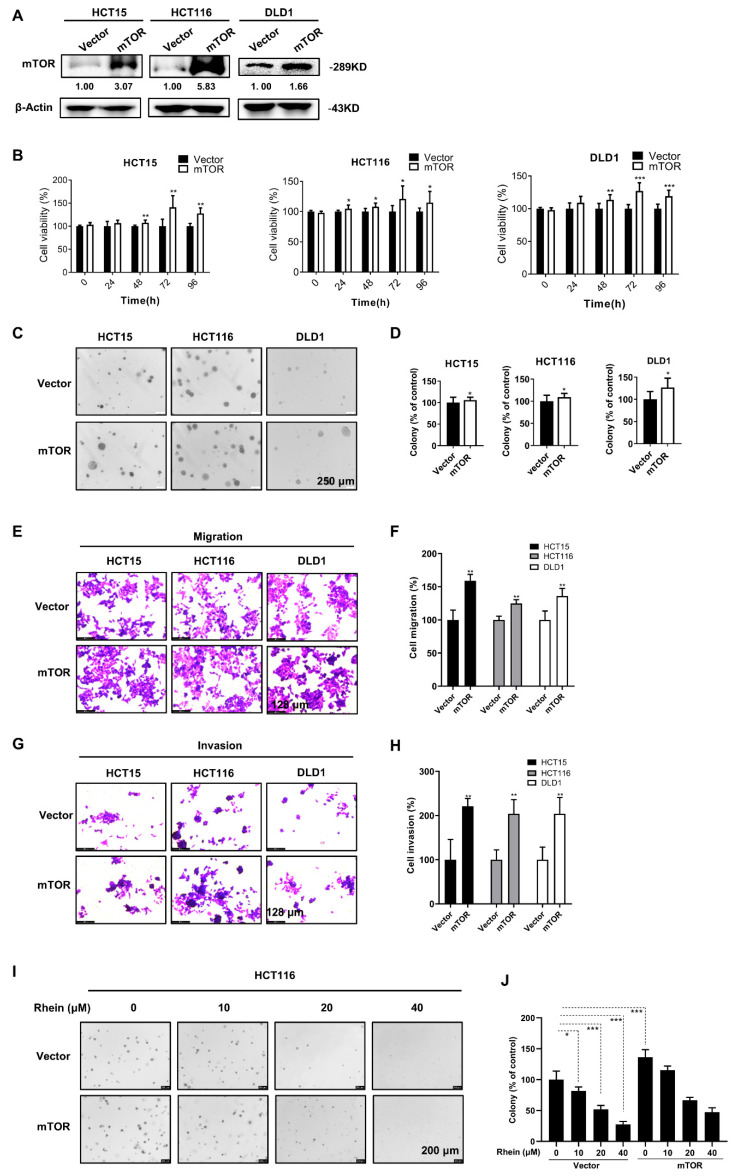
Overexpression of mTOR promotes the proliferation, anchorage-independent colony formation, migration, and invasion of CRC cells. (**A**) Overexpression of mTOR in HCT15, HCT116, and DLD1 cell lines was confirmed using Western blot analysis. (**B**) Cell viability was assessed by CCK-8 assay in mTOR overexpressing CRC cells. (**C**,**D**) Anchorage-independent colony formation assays in mTOR-overexpressing and control HCT15, HCT116, and DLD1 cells (Scale bars = 250 μm). (**E**,**G**) Representative images of migrating and invading cells are shown in the vector or mTOR overexpressing HCT15, HCT116, and DLD1 cells (Scale bars = 128 μm). (**F**,**H**) Quantification of the migrating or invading cells. (**I**,**J**) Anchorage-independent colony formation assays in mTOR-overexpressing and control HCT116 cells after treated with rhein (Scale bars = 200 μm). * *p* < 0.05, ** *p* < 0.01, *** *p* < 0.001. Uncropped western blots figures are shown in Figure S2.

For assessing the functional role of mTOR in CRC, we established three stable mTOR-overexpressing CRC cell lines. The results of Western blot analysis confirmed a significant increase in mTOR expression in HCT15, HCT116, and DLD1 cells compared with the control cells (Figure 6A). The results of CCK-8 assays and anchorage-independent colony formation assays indicated that overexpressing mTOR promoted the proliferation and anchorage-independent colony formation of CRC cells (Figure 6B–D). Moreover, overexpression of mTOR significantly enhanced the migration and invasion abilities of CRC cells as measured by Transwell assays (Figure 6E–H). In addition, overexpression of mTOR attenuated the effect of rhein in HCT116 colony formation ability (Figure 6I,J).

### 3.7. Knockdown of mTOR Suppresses the Proliferation, Anchorage-Independent Colony Formation, Migration, and Invasion of CRC Cells

**Figure 7:**
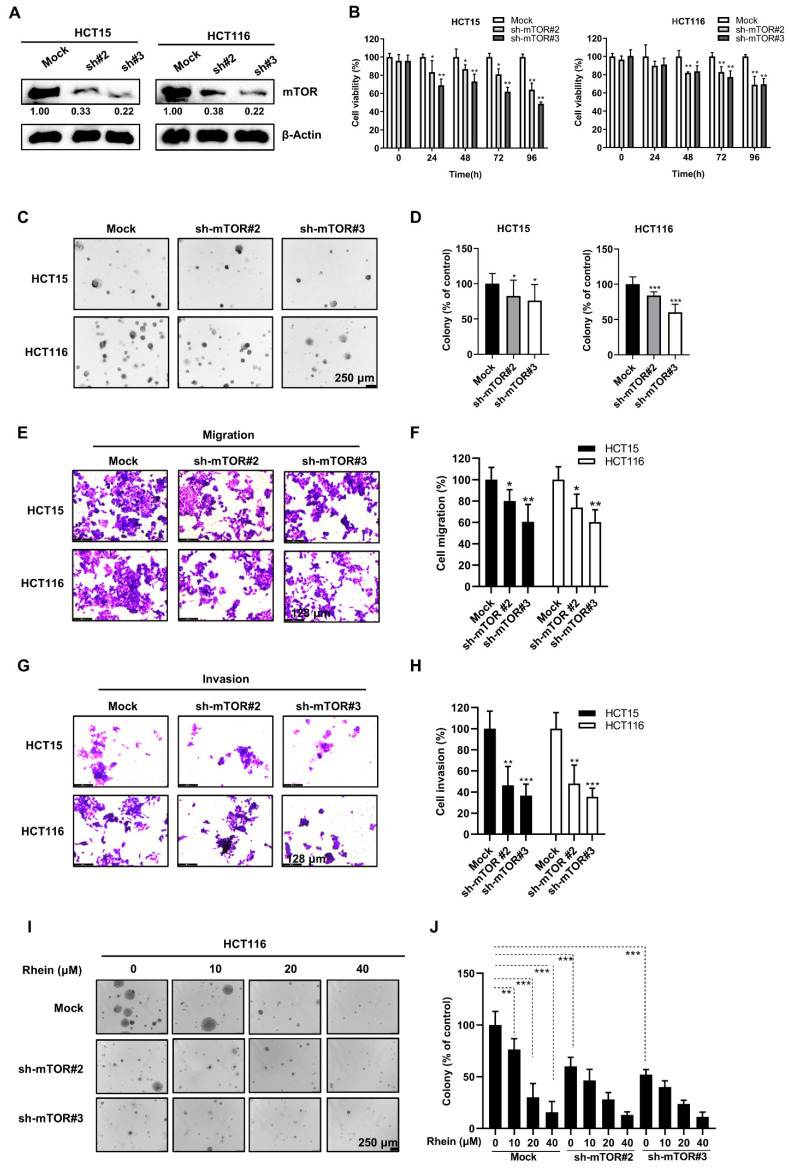
mTOR knockdown suppresses the proliferation, anchorage-independent colony formation, migration, and invasion of CRC cells. (**A**) Western blot analysis showing knockdown of mTOR following shRNA transfection in HCT15 and HCT116 cell lines. (**B**) Cell viability of HCT15 and HCT116 was assessed using the CCK-8 assay. (**C**,**D**) Anchorage-independent colony formation assays in mTOR knockdown and control HCT15 and HCT116 cells (Scale bars = 250 μm). (**E**,**G**) Representative images of migrating and invading cells are shown in HCT15 and HCT116 cells (Scale bars = 128 μm). (**F**,**H**) Quantification of the migrating and invading cells. (**I**,**J**) Anchorage-independent colony formation assays in mTOR knockdown and control HCT116 cells after treated with rhein (Scale bars = 250 μm). * *p* < 0.05, ** *p* < 0.01, *** *p* < 0.001. Uncropped western blots figures are shown in Figure S2.

To further assess the functional role of mTOR in CRC, we knocked down mTOR in CRC cells using a lentiviral vector carrying shRNA specifically targeting mTOR (Figure 7A). Cell proliferation and anchorage-independent colony formation were significantly inhibited after downregulating mTOR expression (Figure 7B–D). However, compared to mock cells, treatment with the same concentrations of rhein (20, 40 μM) failed to further reduce the colony numbers in the mTOR-knockdown cells (Figure 7I,J), indicating that mTOR is the primary target of rhein during CRC cell proliferation. Furthermore, cell migration and invasion were suppressed in mTOR-knockdown cells compared with control cells (Figure 7E–H). 

### 3.8. Rhein Suppresses HCT116 CRC Tumor Growth in a Xenograft Mouse Model

**Figure 8:**
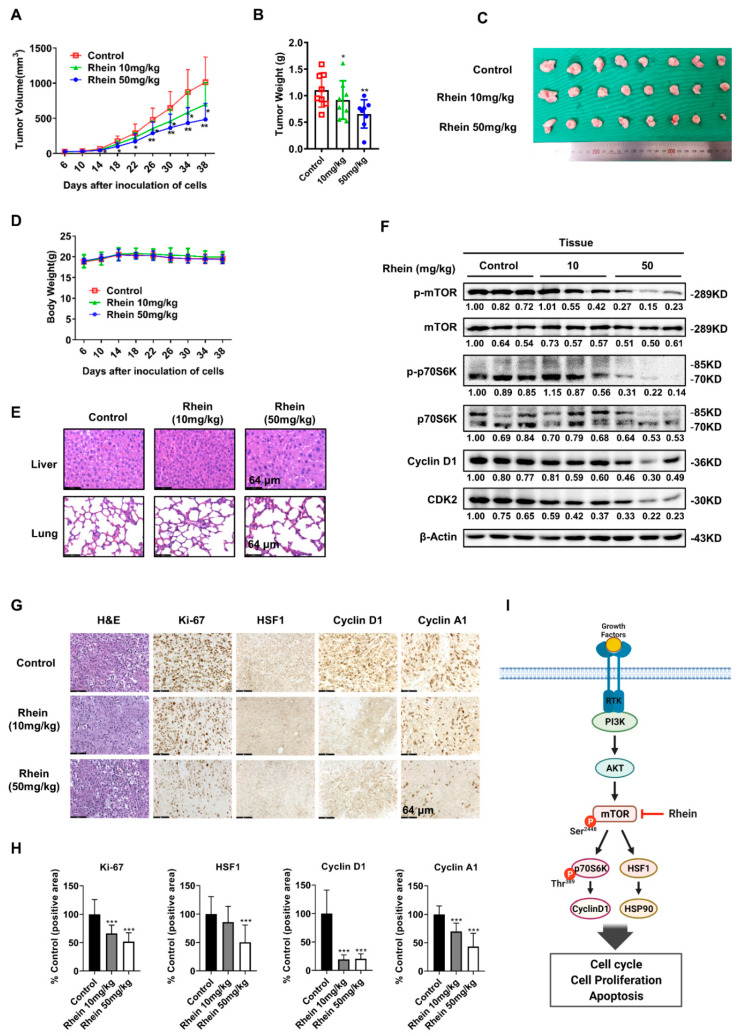
Rhein suppresses HCT116 CRC tumor growth in a xenograft mouse model. (**A**) Nude mice were injected subcutaneously with HCT116 cells. Tumor volumes were plotted over 38 days after inoculation. (**B**) Tumor weights. (**C**) The images show tumors from mice treated with vehicle or rhein (10 or 50 mg/kg). (**D**) Body weights. (**E**) Histopathology conducted by H&E staining of liver and lung samples. (**F**) The expression of p-mTOR, mTOR, p-p70S6K, p70S6K, HSF1, HSP90, cyclin D1, CDK2 and β-Actin was detected by Western blot analysis. (**G**,**H**) Immunohistochemical staining of Ki-67, cyclin D1, HSF1, and cyclin A1 in HCT116 xenograft tumors (Scale bars = 64 μm). (**I**) Schematic diagram of the mechanism of action of rhein based on this study. * *p* < 0.05, ** *p* < 0.01, *** *p* < 0.001. Uncropped western blots figures are shown in Figure S2.

To evaluate the role of rhein on tumor growth in vivo, we established an HCT116 xenografts model by injecting HCT116 cells subcutaneously into the flanks of nude mice to initiate tumor formation. Tumor-bearing mice were divided into three groups and intraperitoneally injected with 2 doses of rhein (10 and 50 mg/kg) or vehicle 3 times a week for 32 days. We observed that treatment with rhein (10 or 50 mg/kg body weight) significantly inhibited tumor growth compared with the vehicle-treated mice (Figure 8A–C). Furthermore, the weight of the mice was monitored every four days and was not affected by rhein administration (Figure 8D). We examined the histological structure of the liver and heart after 32 days of rhein treatment in tumor-bearing mice. The results showed that the histological structure of the liver and lung exhibited no significant changes compared with the vehicle group (Figure 8E). These results demonstrated that rhein could effectively inhibit tumor growth without exhibiting obvious toxicity. To further confirm whether the results of in vitro experiments were consistent with those in vivo, tumor tissues of tumor-bearing mice were collected for western blot analysis and immunohistochemical (IHC) staining. The results indicated that the expression of p-mTOR, p-p70S6K, p70S6K, cyclin D1, and CDK2 was significantly downregulated after treatment with rhein (Figure 8F). In addition, IHC results showed that protein levels of Ki-67, HSF1, cyclin D1, and cyclin A1 were downregulated following treatment with rhein, which was consistent with the in vitro data (Figure 8G,H). Overall, these results demonstrate that rhein effectively inhibits CRC tumor growth through the mTOR pathway in vivo and has potential as a chemotherapeutic agent for CRC. A schematic diagram of the mechanism of action of rhein based on these findings is shown in Figure 8I.

## 4. Discussion

Accumulating evidence indicates that traditional Chinese medicine offers significant advantages in improving the quality of life and prolonging survival of cancer patients with minimal toxicity. Chinese medicinal formulas, herbs, and their active ingredients have attracted increased attention from cancer researchers [40,41,42]. Studies have demonstrated that rhein shows potent efficacy in inhibiting tumor growth [19,24]. However, the underlying mechanism(s) of rhein in CRC remain poorly understood. In this study, we demonstrated that rhein suppresses CRC cell growth by inducing S-phase cell cycle arrest and apoptosis, and suppresses CRC cell migration and invasion by inhibiting EMT. A mechanistic study revealed that rhein exerts anticancer activity by promoting ubiquitin-mediated mTOR degradation and suppressing mTOR signaling in vitro and in vivo.

The mTOR signaling pathway plays a central role in cell proliferation and metabolism and is involved in tumor initiation and progression [43,44]. Inhibition of mTOR was shown to effectively suppress tumor growth in CRC [9,45]. mTORC1 and mTORC2 are two different mTOR complexes. mTORC1 phosphorylates p70S6K and eukaryotic initiation factor 4E-binding protein1 (4E-BP1) leading to increased cell proliferation [46]. mTORC2 phosphorylates Akt at Ser 473 leading to activation of the Akt signaling pathway [47]. A previous study demonstrated that mTOR is essential for HSF1 activation and heat-shock protein synthesis [35]. Recent studies have shown that HSF1 is associated with advanced tumor progression and poor prognosis in gastric cancer [48] and breast cancer [49]. In this study, we found that mTOR was highly expressed in CRC patient tumor tissues (Figure 4A) and cells (Figure 4B). Treatment with rhein significantly suppressed mTOR and the expression of its downstream effectors, p70S6K and 4EBP1, in CRC cells (Figure 4D) and xenograft tumor tissues (Figure 8F). We also found that HSF1 was highly expressed in CRC cells and rhein treatment downregulated the expression of HSF1 and HSP90. These findings indicate that rhein inhibits both the mTOR/p70S6K and mTOR/HSF1 pathways. We further demonstrated that rhein downregulates mTOR expression by promoting mTOR ubiquitination which leads to mTOR degradation (Figure 5).

Cell cycle progression is monitored strictly by CDKs and their partner cyclins [50]. The cyclin A/CDK2 complex is required for progression through the S-phase [51]. S-phase cell cycle arrest was observed in cells exposed to hypoxia [52], DNA damage [53], and chemotherapy [54]. The mTOR pathway has been reported to be involved in the DNA damage response [55]. Interestingly, our results showed rhein-induced S-phase cell cycle arrest through downregulation of cyclin A1, cyclin E1, and CDK2 in CRC cells. Whether rhein can induce CRC cell DNA damage will need to be confirmed in a future study.

Cancer metastasis is a major cause of treatment failure. The EMT is a key mechanism involved in cancer metastasis [56]. During EMT, epithelial cells transform into migrating and infiltrating cells. Cancer cells appear to lose epithelial markers, such as E-cadherin, and acquire mesenchymal markers, such as N-cadherin [57]. In the present study, we found that rhein significantly suppressed the migration and invasion of CRC cells accompanied by downregulated expression of N-cadherin and vimentin and upregulated expression of E-cadherin, indicating that rhein can suppress the EMT process (Figure 2). In addition, our results indicated that mTOR plays an important role in the EMT process. Overexpression of mTOR promoted CRC cell migration and invasion (Figure 6), whereas mTOR knockdown inhibited CRC cell migration and invasion (Figure 7).

Cell line-derived tumor xenograft models are commonly used for assessing cancer therapeutic efficacy. To further confirm the antitumor effects of rhein, we established an HCT116 xenograft mouse model in vivo. Our results shown that rhein markedly suppressed CRC tumor growth without causing toxicity based on insignificant loss of body weight and histological lesions of liver or lung tissues compared with the vehicle group mice (Figure 8D,E). Rhein treatment also downregulated p-mTOR, p-p70S6K, HSF1, and cyclin D1 in tumor tissues, which further confirmed that rhein suppressed CRC cell growth by inhibiting the mTOR signaling pathway. 

## 5. Conclusions

In summary, we demonstrated that rhein inhibited CRC cell growth in vitro and in vivo by directly targeting mTOR and suppressing the mTOR signaling pathway, which plays a vital role in the progression of CRC. Our study indicates that rhein may be a potential antitumor agent for CRC prevention and treatment.

## Data Availability

Data is contained within the Results and Supplementary Materials.

## Supplementary Materials

The following are available online at https://www.mdpi.com/article/10.3390/cancers13092176/s1, Figure S1: Effect of rhein on the viability of CRC cells and normal colon fibroblasts cells, Figure S2: Original, uncropped blots.
