# Supplementary material for: Rhein Suppresses Colorectal Cancer Cell Growth by Inhibiting the mTOR Pathway In Vitro and In Vivo
Source: Cancers (Basel). 2021 Apr 30;13(9):2176. doi: 10.3390/cancers13092176 (PMC8125196; doi:10.3390/cancers13092176)
Supplement: Supplementary file 1 [file cancers-13-02176-s001.zip › Figure S1.pdf]

Supplementary Figure

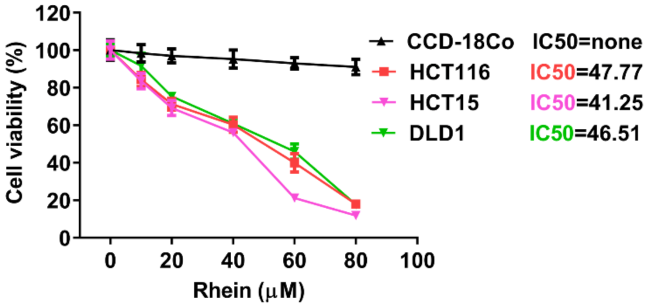

Figure S1. Effect of rhein on the viability of CRC cells and normal colon fibroblasts cells. HCT15, HCT116, DLD1, and CCD-18Co cells were treated with rhein (0, 10, 20, 40, 60, and 80 μM) for 24 h. Cell viability was determined by CCK-8 assay.
