# Supplementary material for: Rhein Suppresses Colorectal Cancer Cell Growth by Inhibiting the mTOR Pathway In Vitro and In Vivo
Source: Cancers (Basel). 2021 Apr 30;13(9):2176. doi: 10.3390/cancers13092176 (PMC8125196; doi:10.3390/cancers13092176)
Supplement: Supplementary file 1 [file cancers-13-02176-s001.zip › Figure S2 Original, uncropped blots.pdf]

Figure 2G

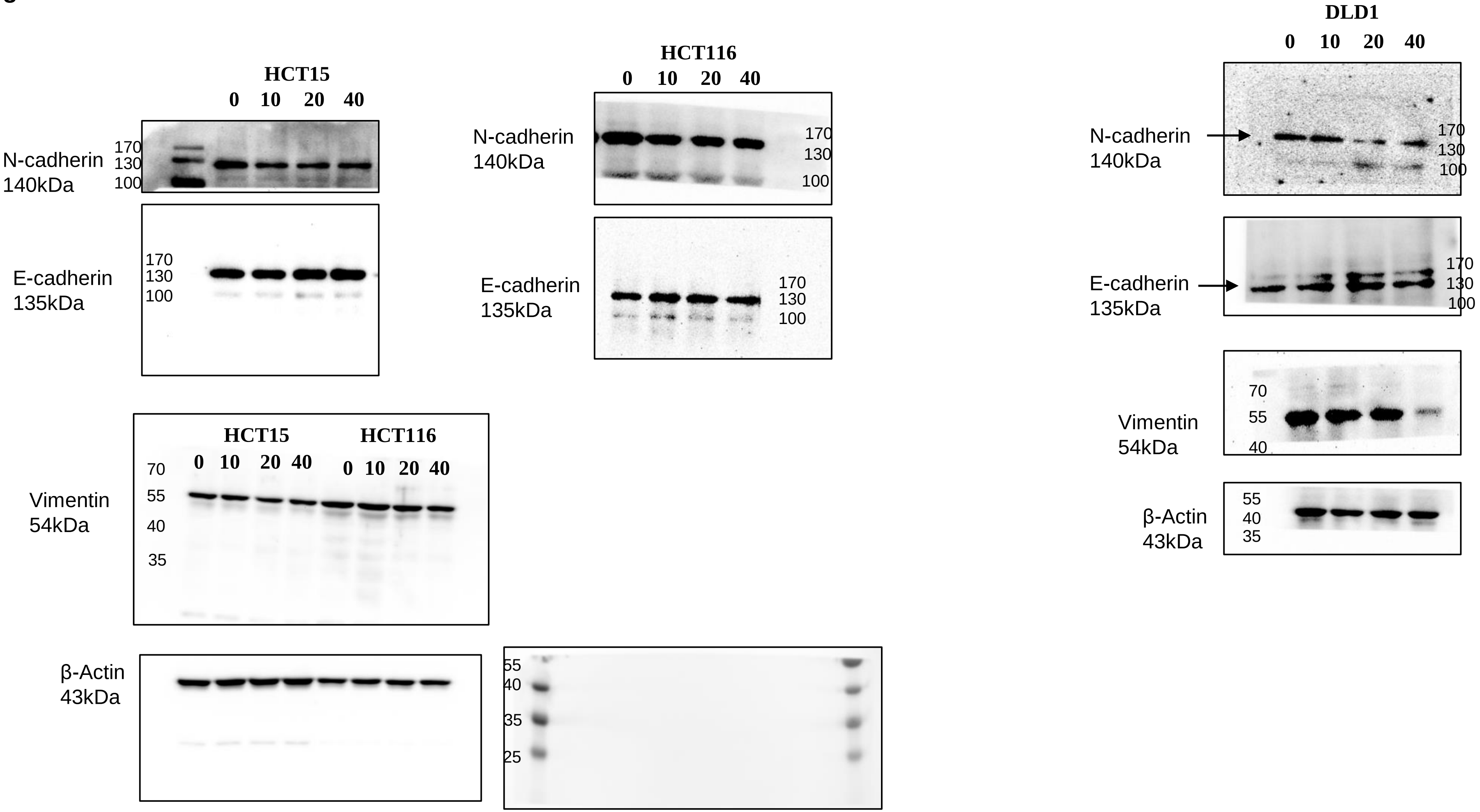
HCT15
0 10 20 40
N-cadherin
140kDa
170
130
100
E-cadherin
135kDa
170
130
100
HCT116
0 10 20 40
N-cadherin
140kDa
170
130
100
E-cadherin
135kDa
170
130
100
DLD1
0 10 20 40
N-cadherin
140kDa
170
130
100
E-cadherin
135kDa
170
130
100
Vimentin
54kDa
70
55
40
β-Actin
43kDa
55
40
35
HCT15
HCT116
0 10 20 40
0 10 20 40
Vimentin
54kDa
70
55
40
35
β-Actin
43kDa
55
40
35
25

Figure 3C

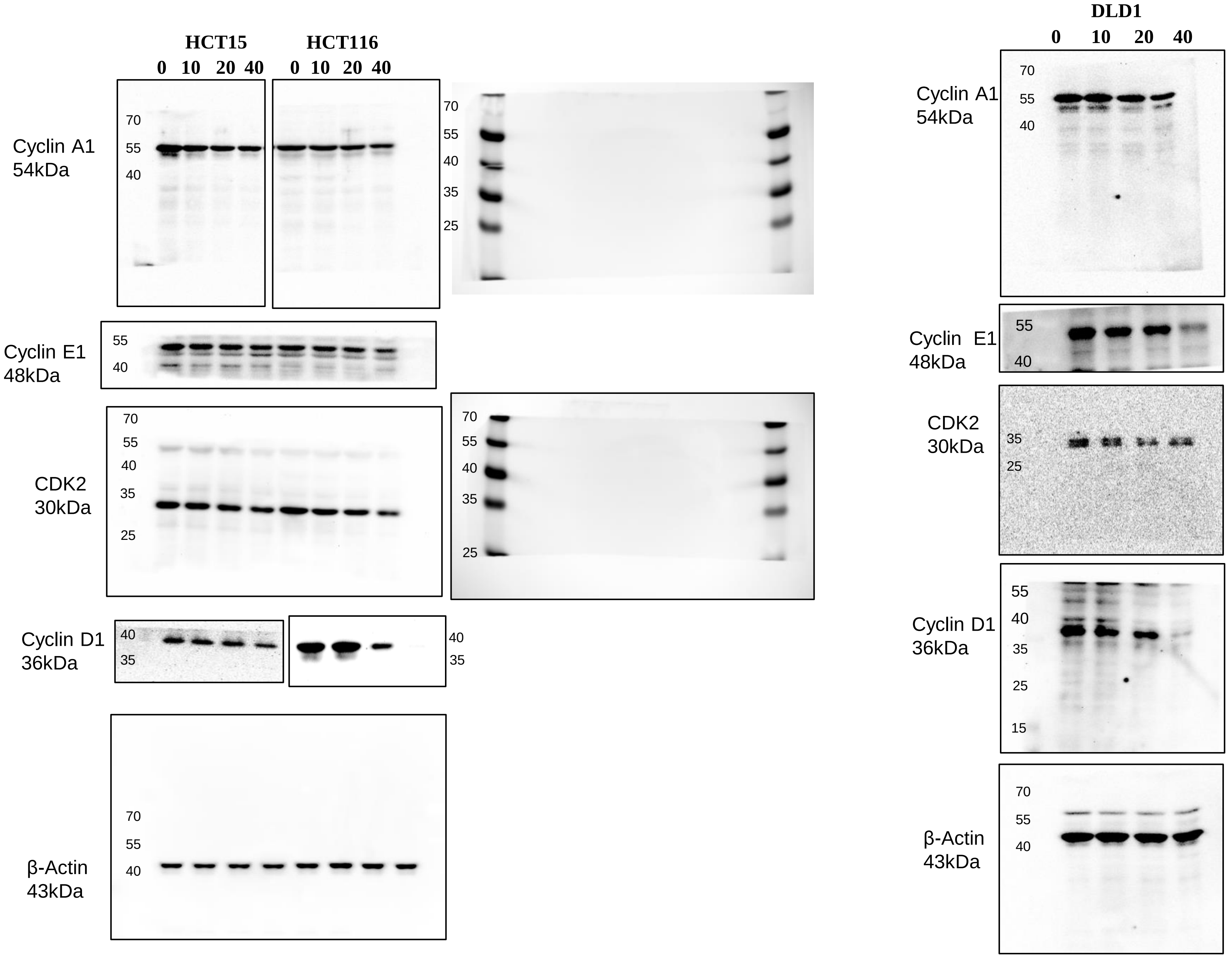
HCT15
HCT116
0 10 20 40 0 10 20 40
Cyclin A1
54kDa
70
55
40
35
25
Cyclin E1
48kDa
CDK2
30kDa
Cyclin D1
36kDa
β-Actin
43kDa
DLD1
0 10 20 40
15

Figure 3F

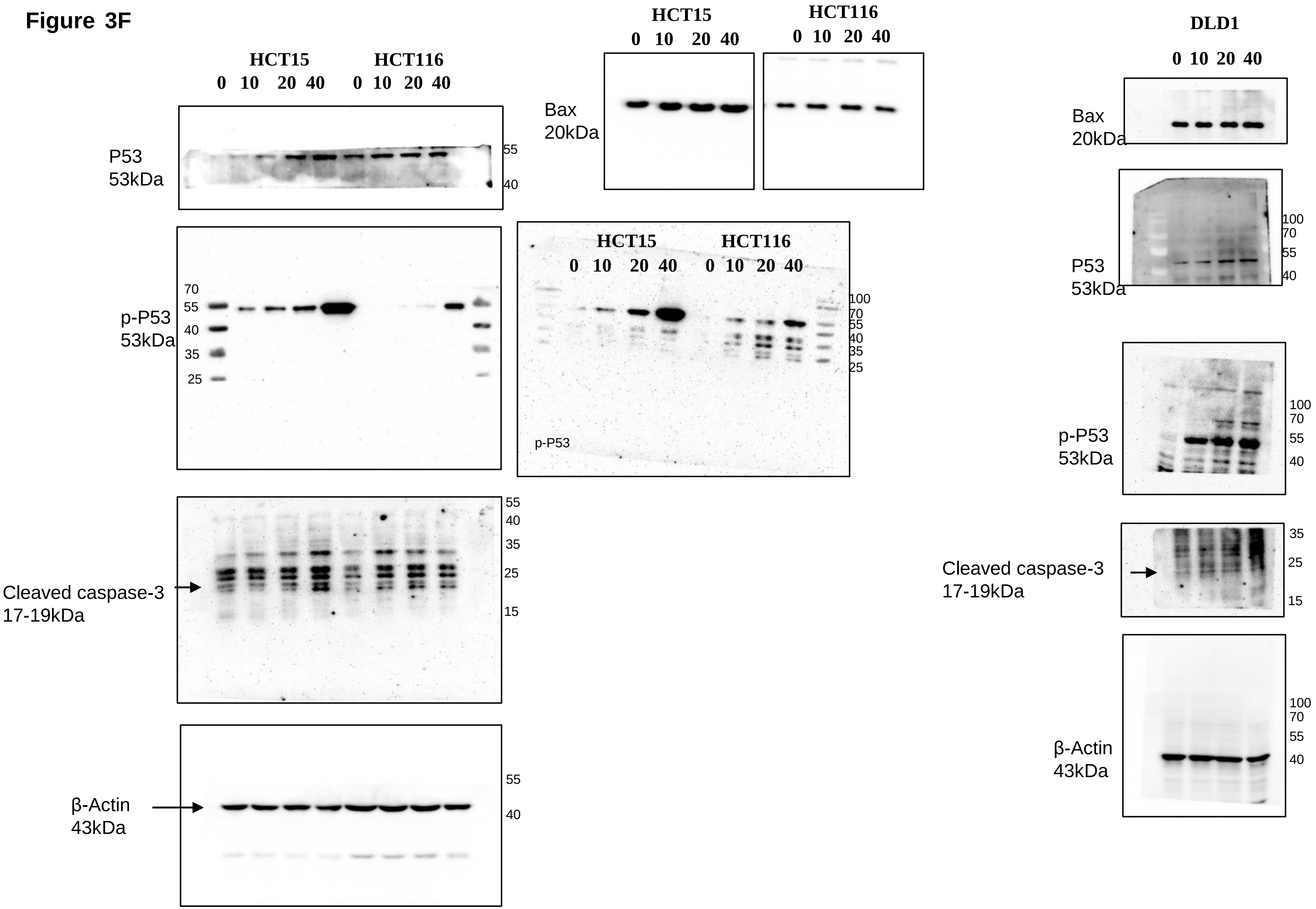

HCT15
HCT116
0 10 20 40 0 10 20 40
P53
53kDa
p-P53
53kDa
Cleaved caspase-3
17-19kDa
β-Actin
43kDa
Bax
20kDa
DLD1
0 10 20 40
100
70
55
40
35
25
15

Figure 4B

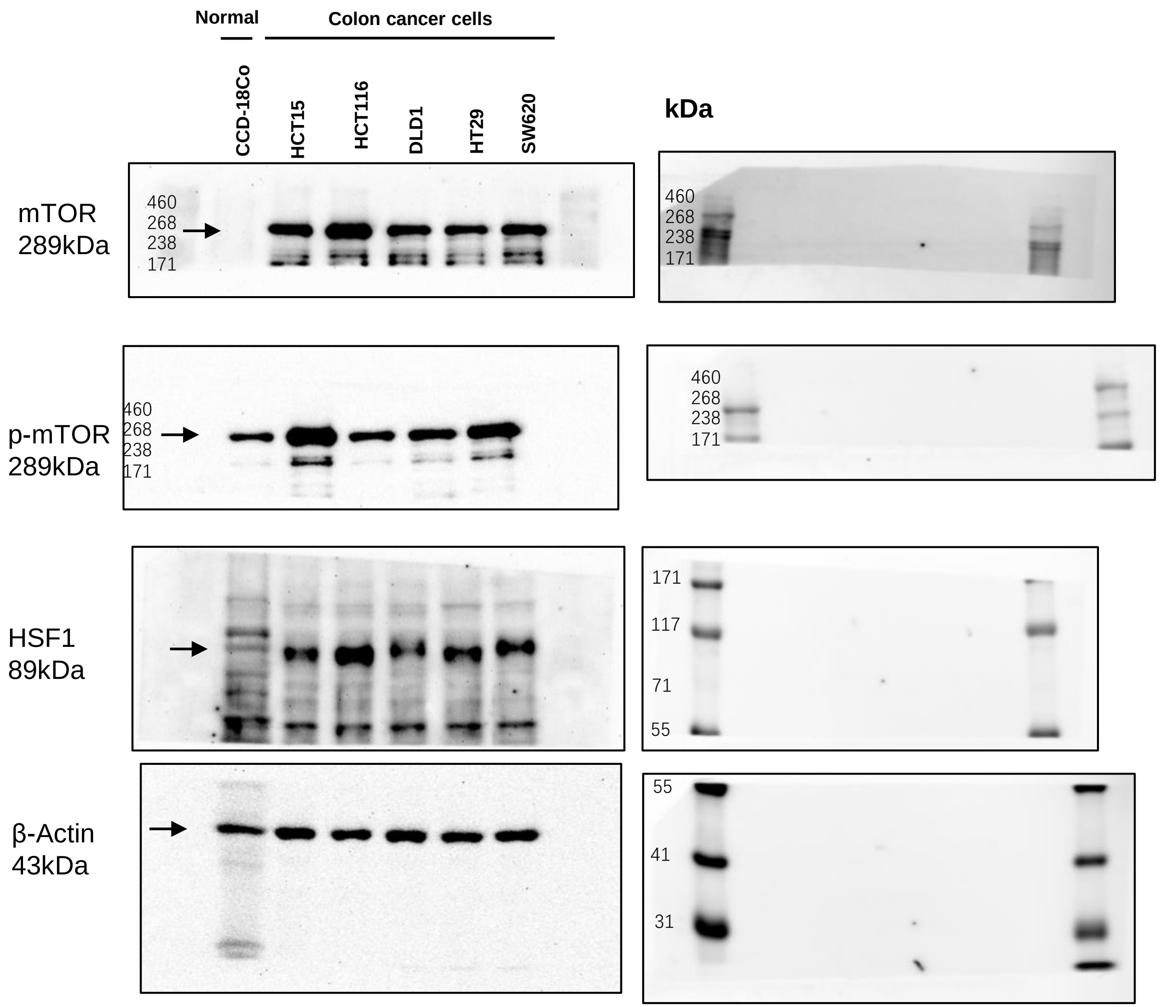

Figure 4C

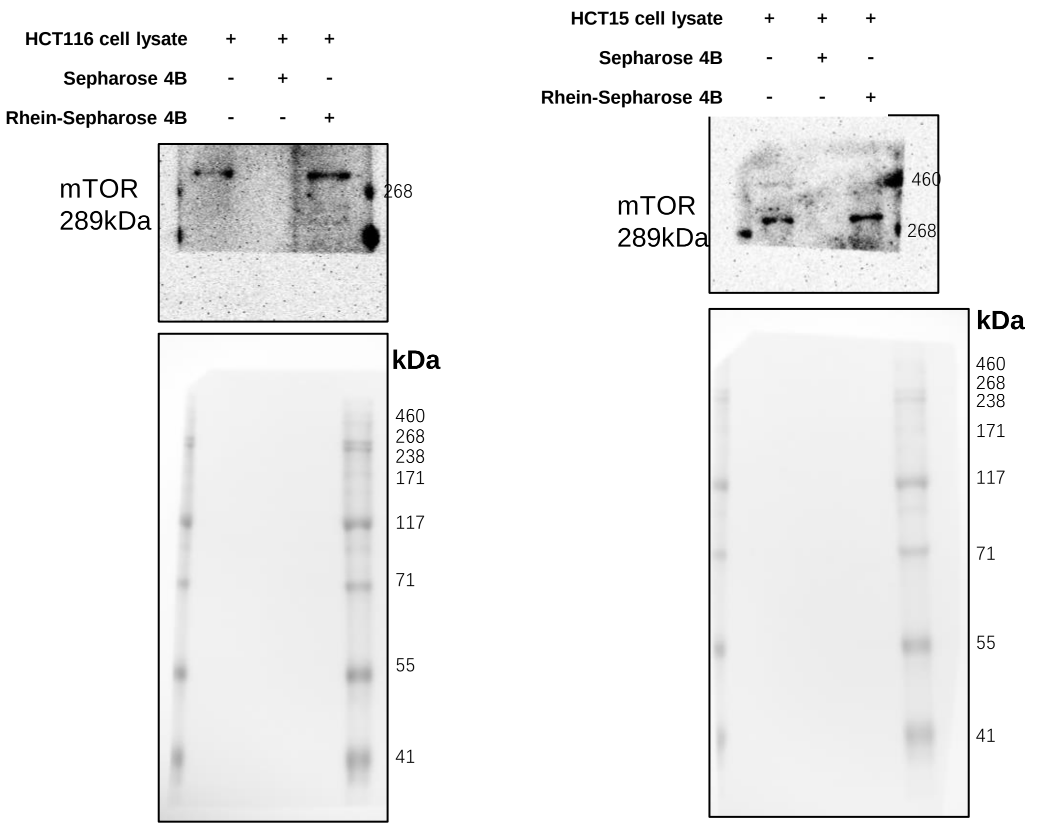

Figure 4D

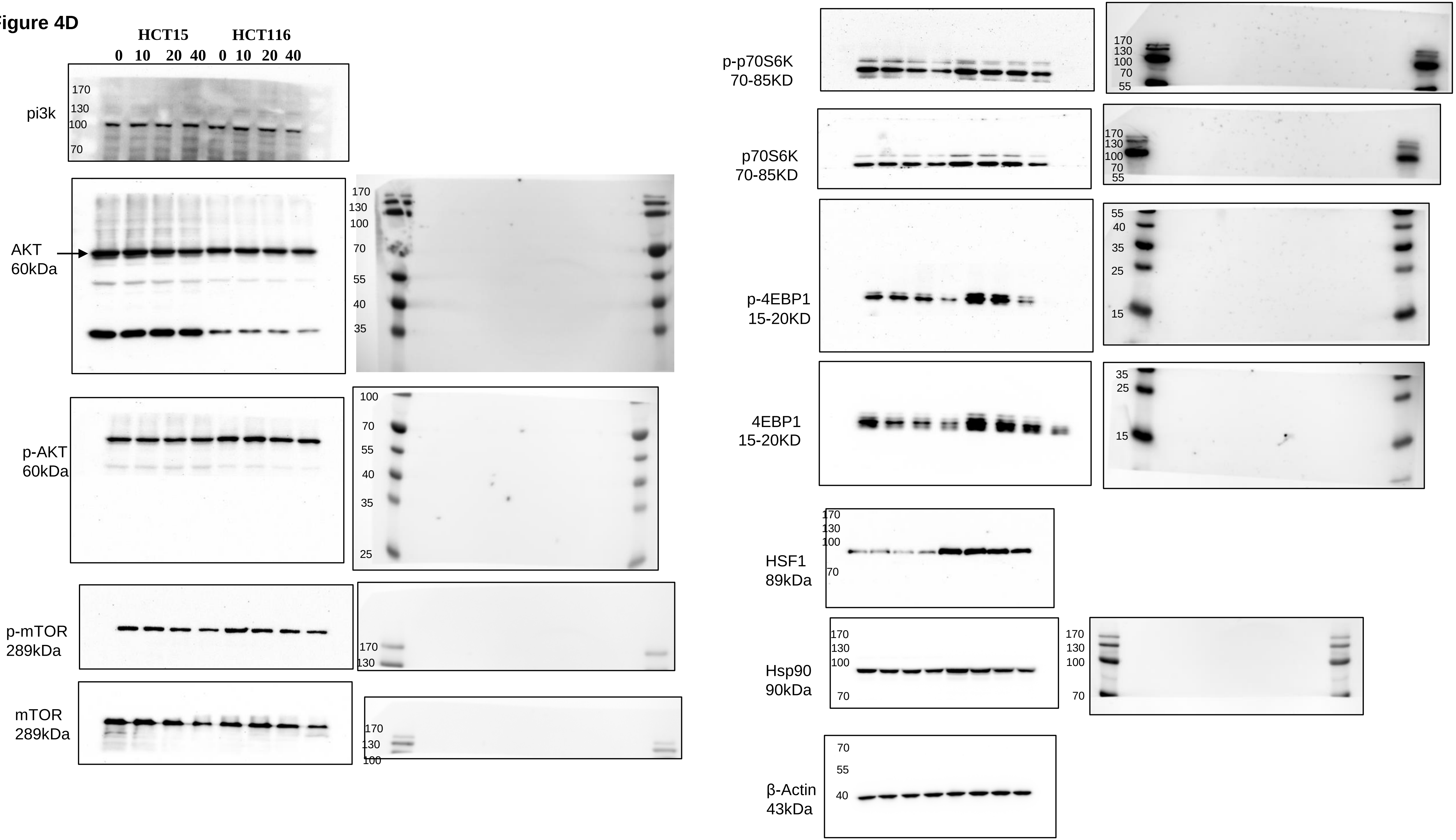
HCT15
HCT116
0 10 20 40 0 10 20 40
pi3k
170
130
100
70
AKT
60kDa
170
130
100
70
55
40
35
p-AKT
60kDa
100
70
55
40
35
25
p-mTOR
289kDa
170
130
mTOR
289kDa
170
130
100
p-p70S6K
70-85KD
170
130
100
70
55
p70S6K
70-85KD
170
130
100
70
55
p-4EBP1
15-20KD
55
40
35
25
15
4EBP1
15-20KD
35
25
15
HSF1
89kDa
170
130
100
70
Hsp90
90kDa
170
130
100
70
170
130
100
70
β-Actin
43kDa
70
55
40

Figure 5A

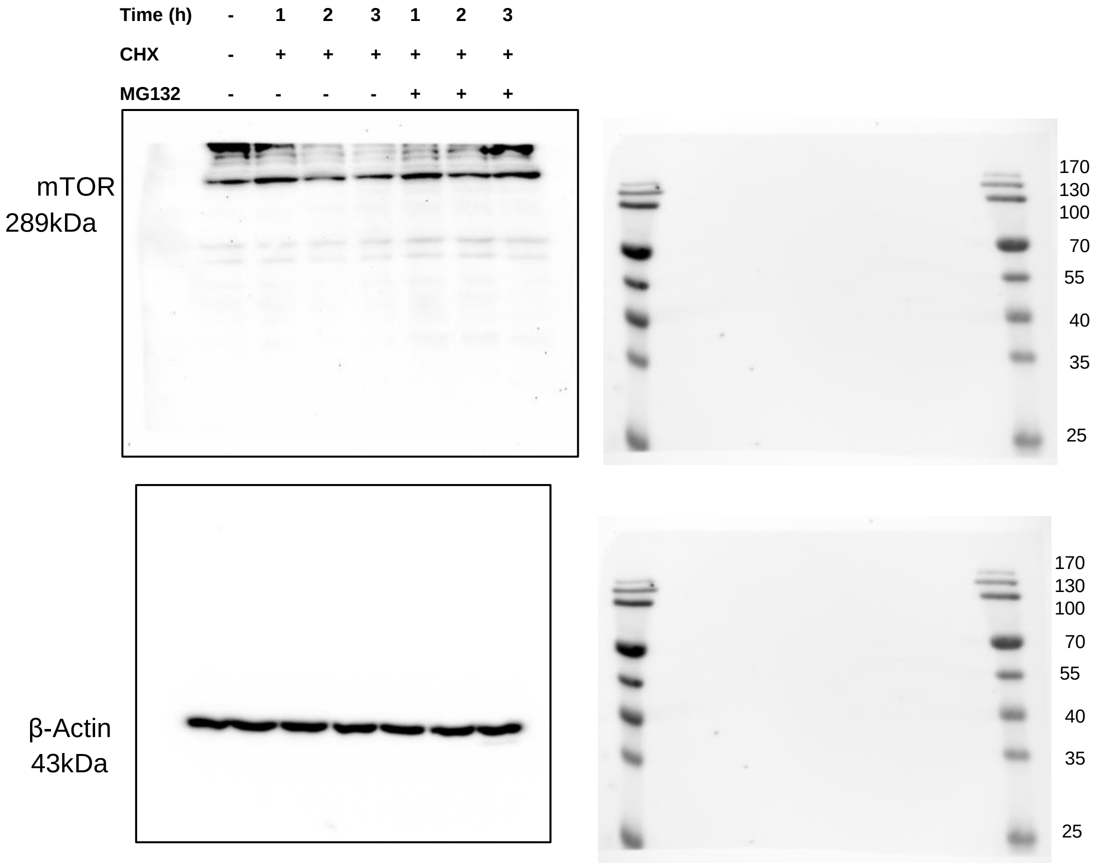

Figure 5B

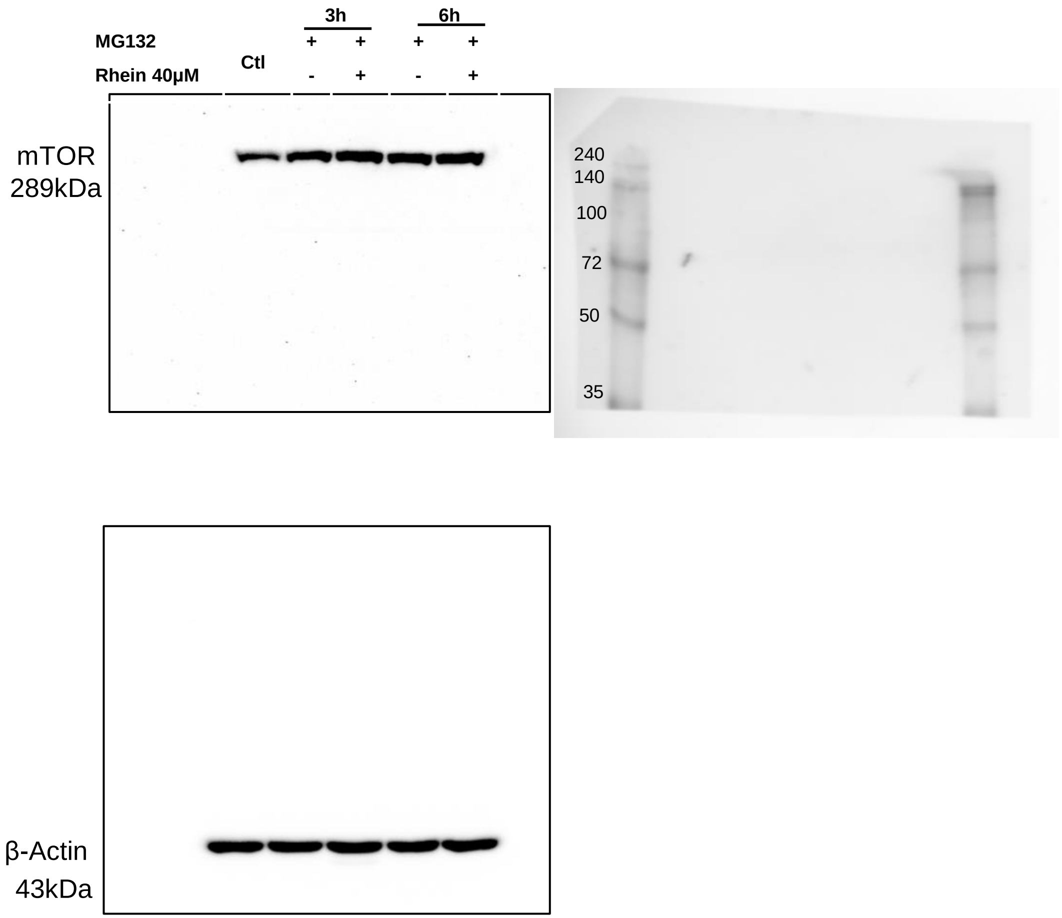

Figure 5C

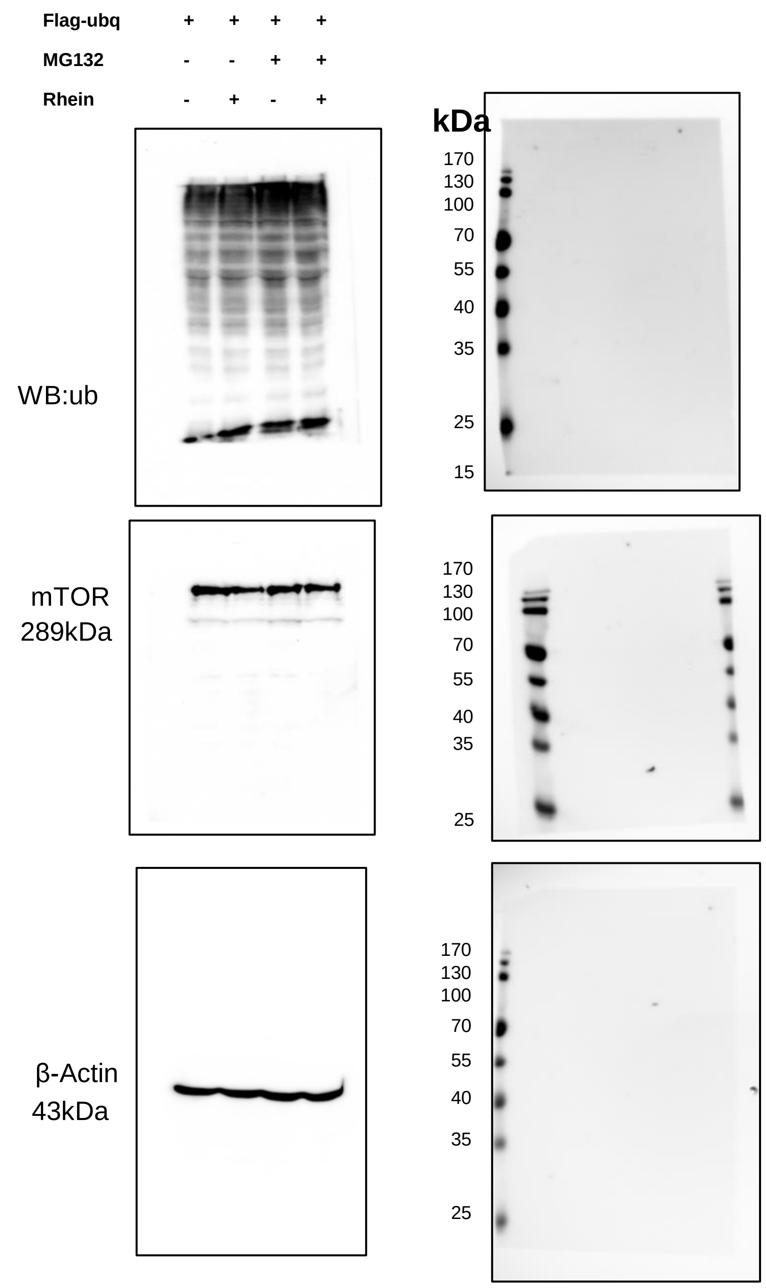

Figure 5D

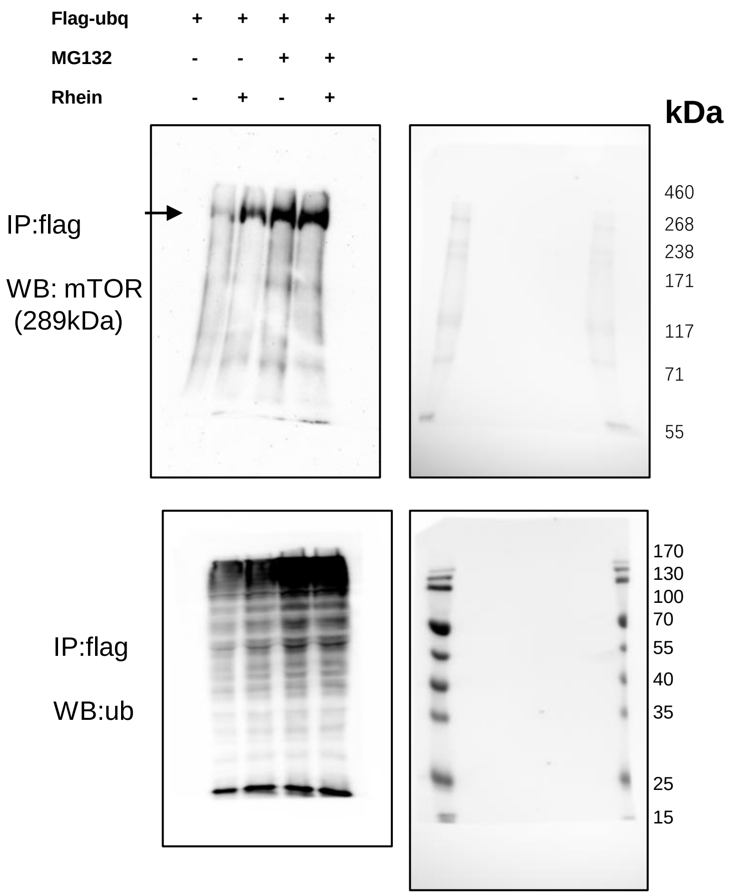

Figure 6A

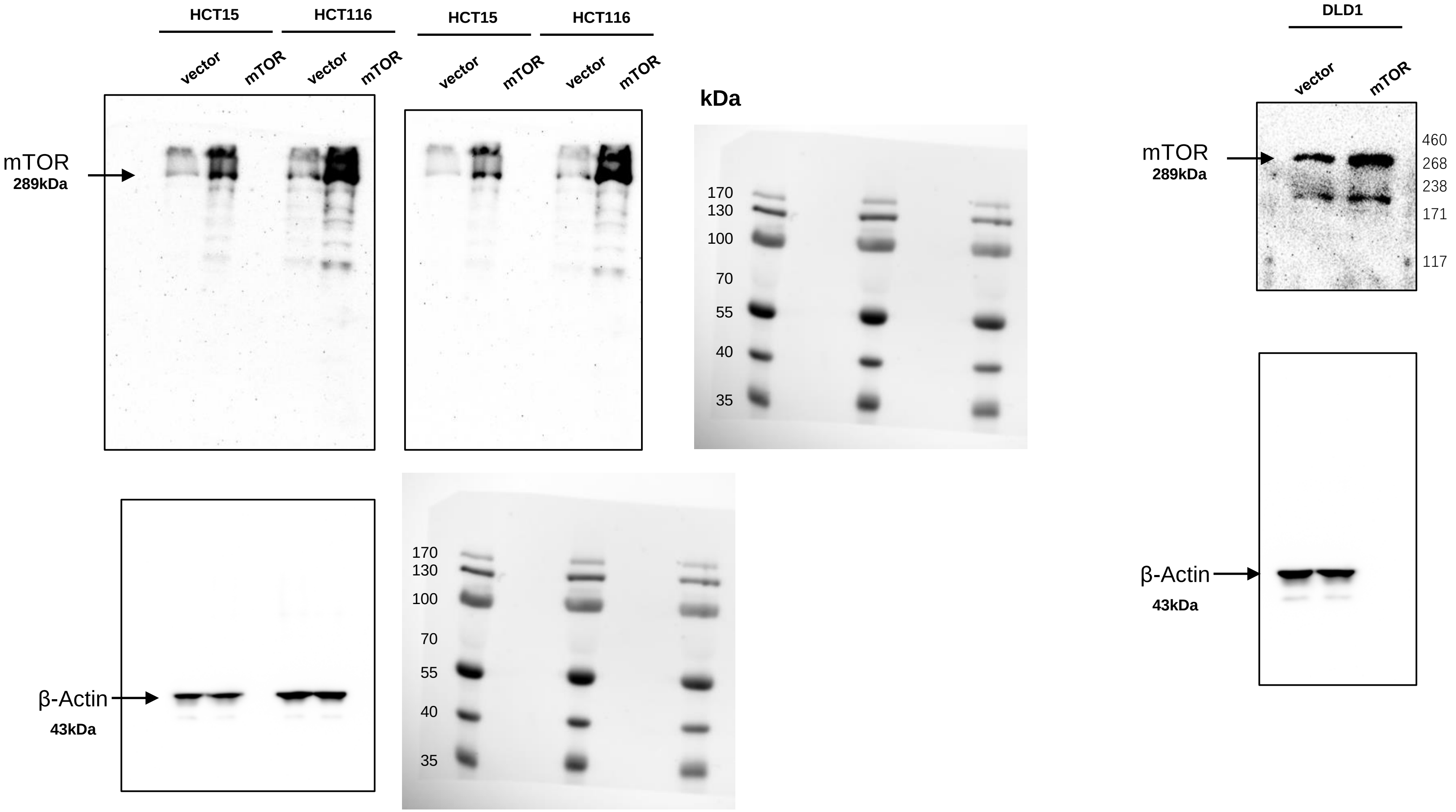

HCT15
HCT116
vector
mTOR
vector
mTOR
HCT15
HCT116
vector
mTOR
vector
mTOR
kDa
mTOR
289kDa
170
130
100
70
55
40
35
β-Actin
43kDa
170
130
100
70
55
40
35
DLD1
vector
mTOR
mTOR
289kDa
460
268
238
171
117
β-Actin
43kDa

Figure 7A

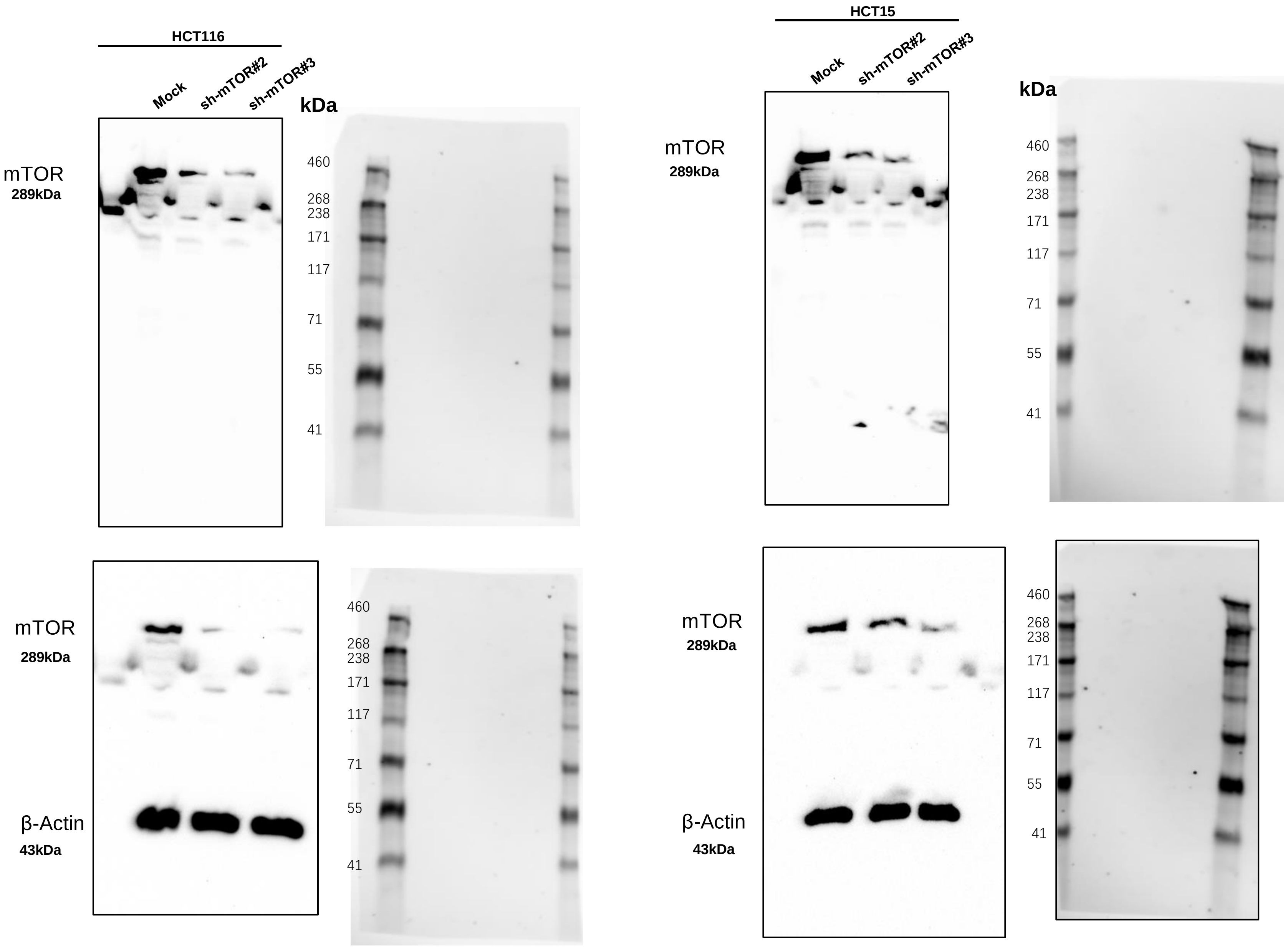
HCT116
Mock
sh-mTOR#2
sh-mTOR#3
kDa
mTOR
289kDa
460
268
238
171
117
71
55
41
mTOR
289kDa
β-Actin
43kDa
HCT15
mTOR
289kDa
β-Actin
43kDa

Figure 8F

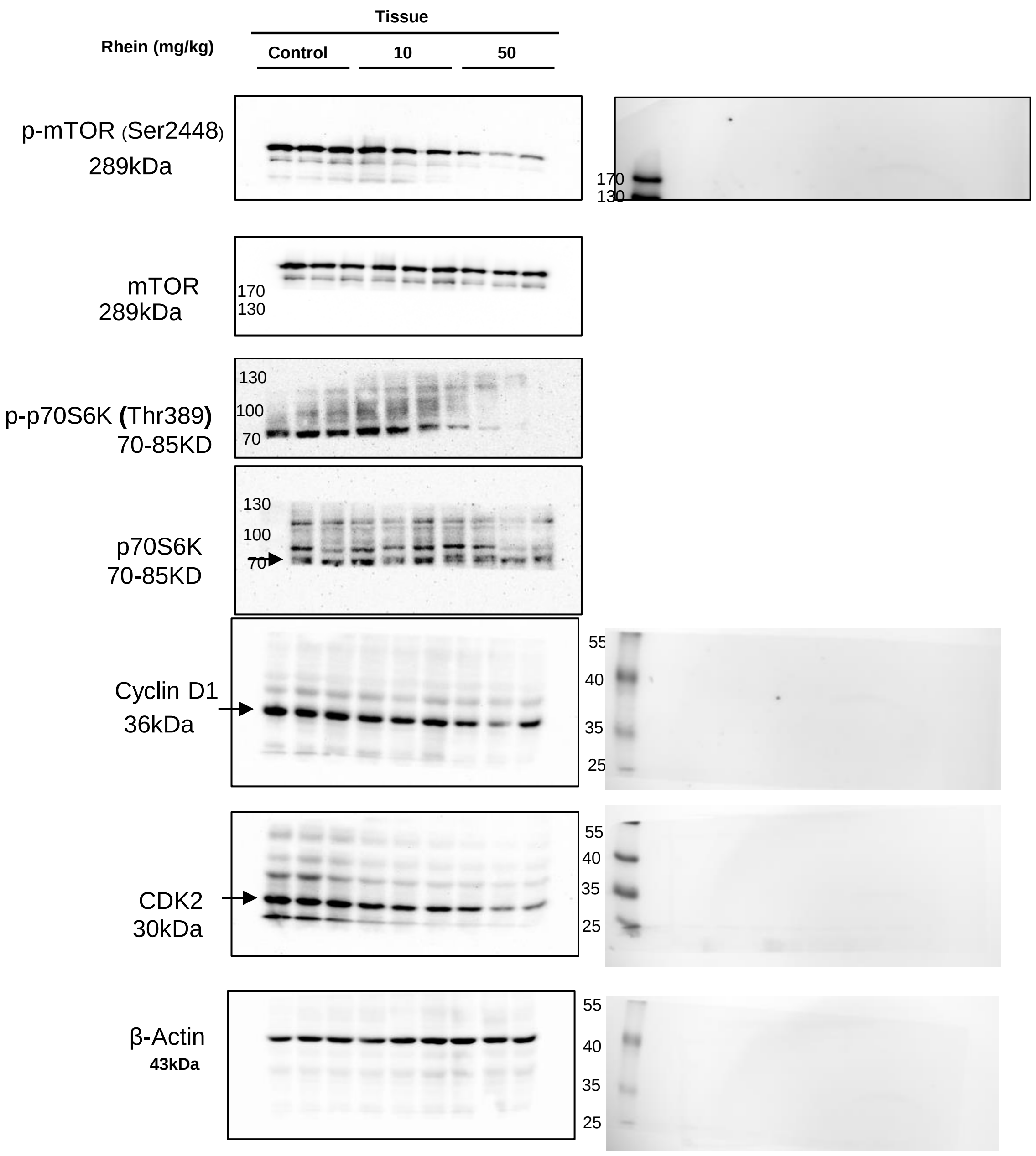

Tissue
Rhein (mg/kg)
Control
10
50
p-mTOR (Ser2448)
289kDa
170
130
mTOR
289kDa
170
130
p-p70S6K (Thr389)
70-85KD
130
100
70
p70S6K
70-85KD
130
100
70
Cyclin D1
36kDa
55
40
35
25
CDK2
30kDa
55
40
35
25
β-Actin
43kDa
55
40
35
25
